# Effects of Ganjianglingzhu Decoction on Lean Non-Alcoholic Fatty Liver Disease in Mice Based on Untargeted Metabolomics

**DOI:** 10.3390/ph17040502

**Published:** 2024-04-15

**Authors:** Nan Tang, Lei Ji, Xinyu Shi, Yalan Xiong, Xinying Xiong, Hanhua Zhao, Hualing Song, Jianying Wang, Lei Zhang, Shengfu You, Guang Ji, Baocheng Liu, Na Wu

**Affiliations:** 1 School of Public Health, Shanghai Innovation Center of Traditional Chinese Medicine Health Service, Shanghai University of Traditional Chinese Medicine, Shanghai 201203, China; tangnan@shutcm.edu.cn (N.T.); 22022547@shutcm.edu.cn (X.S.); yalanxiongg@shutcm.edu.cn (Y.X.); 0022023573@shutcm.edu.cn (X.X.); 99shl@163.com (H.S.); wjy8310@163.com (J.W.); zhanglei37@sina.com (L.Z.); jg@shutcm.edu.cn (G.J.); 2 Bio-X Institutes, Key Laboratory for the Genetics of Developmental and Neuropsychiatric Disorders, Shanghai Jiao Tong University, Shanghai 200030, China; amber_jl@sjtu.edu.cn; 3 Department of Sport Science, College of Education, Zhejiang University, Hangzhou 310058, China; 12003024@zju.edu.cn; 4 Institute of Digestive Diseases, Longhua Hospital, Shanghai University of Traditional Chinese Medicine, Shanghai 200032, China; yoosf@sina.com; 5 State Key Laboratory of Integration and Innovation of Classic Formula and Modern Chinese Medicine, Shanghai University of Traditional Chinese Medicine, Shanghai 201203, China

**Keywords:** Ganjianglingzhu decoction, lean NAFLD, glycerophospholipid metabolism

## Abstract

Non-alcoholic fatty liver disease (NAFLD) is usually associated with obesity. However, it is crucial to recognize that NAFLD can also occur in lean individuals, which is frequently overlooked. Without an approved pharmacological therapy for lean NAFLD, we aimed to investigate whether the Ganjianglingzhu (GJLZ) decoction, a representative traditional Chinese medicine (TCM), protects against lean NAFLD and explore the potential mechanism underlying these protective effects. The mouse model of lean NAFLD was established with a methionine-choline-deficient (MCD) diet in male C57BL/6 mice to be compared with the control group fed the methionine-choline-sufficient (MCS) diet. After four weeks, physiological saline, a low dose of GJLZ decoction (GL), or a high dose of GJLZ decoction (GH) was administered daily by gavage to the MCD group; the MCS group was given physiological saline by gavage. Untargeted metabolomics techniques were used to explore further the potential mechanism of the effects of GJLZ on lean NAFLD. Different doses of GJLZ decoction were able to ameliorate steatosis, inflammation, fibrosis, and oxidative stress in the liver; GL performed a better effect on lean NAFLD. In addition, 78 candidate differential metabolites were screened and identified. Combined with metabolite pathway enrichment analysis, GL was capable of regulating the glucose and lipid metabolite pathway in lean NAFLD and regulating the glycerophospholipid metabolism by altering the levels of sn-3-O-(geranylgeranyl)glycerol 1-phosphate and lysoPC(P-18:0/0:0). GJLZ may protect against the development of lean NAFLD by regulating glucose and lipid metabolism, inhibiting the levels of sn-3-O-(geranylgeranyl)glycerol 1-phosphate and lysoPC(P-18:0/0:0) in glycerophospholipid metabolism.

## 1. Introduction

As the most prevalent chronic liver disease, the overall morbidity of non-alcoholic fatty liver disease (NAFLD) worldwide has significantly increased, from 25.5% to 37.8% in the last 20 years [1]. NAFLD covers a series of illnesses, including liver steatosis and non-alcoholic steatohepatitis (NASH), and leads to liver fibrosis and hepatocellular carcinoma [2]. Increasing evidence has demonstrated NAFLD is closely related to metabolic syndrome, covering type 2 diabetes mellitus, dyslipidemia, obesity, and hypertension [3]. Of note, NAFLD is not exclusive to overweight or obese individuals; it is also discovered in lean people. NAFLD in lean individuals is far from uncommon, with prevalence rates around 14.5%, but it is easier to ignore than NAFLD in overweight or obese individuals, which leads to severe consequences [4]. 

Lean NAFLD represents a proportion of NAFLD cases that have a normal body mass index (BMI); an estimated 13.11% of individuals with NAFLD have lean body habitus [5,6]. The World Health Organization’s standard defines normal weight as a BMI between 18.5 and 24.9 kg/m^2^ [7]. Otherwise, visceral fat accumulation is detrimental to lean NAFLD patients, making waist circumference a supplementary indicator of BMI [8]. Some reports show that lean NAFLD patients exhibit a higher fibrosis-4 index and mortality than obese NAFLD patients [9,10,11]. At present, the pathophysiology of lean NAFLD remains obscure.

The current treatment for lean NAFLD is to lose 3–5% of weight through lifestyle intervention, including exercise and dietary changes [12]. Nevertheless, the majority of patients have difficulty maintaining a lifestyle intervention in practice. Moreover, the lack of effective therapeutic medicine for lean NAFLD remains a serious problem. The current medicines for NAFLD are vitamin E, metformin, glucagon-like peptide-1 agonists (liraglutide), and pioglitazone. Still, their therapeutic role in lean NAFLD needs to be further explored [5,12]. Therefore, there is an urgent need to conduct research into lean NAFLD’s pathogenesis and undertake drug development. 

The active ingredients in a large number of traditional Chinese medicines (TCMs) significantly affect NAFLD [13,14]. Due to the lack of pharmaceutical options in lean NAFLD, we intended to discover a kind of TCM that is effective for lean NAFLD. The Ganjianglingzhu (GJLZ) decoction is a representative formula that successfully alleviates hepatic steatosis and inflammation in NAFLD rats [15,16]. Whether it is also effective against lean NAFLD is unclear. 

Therefore, we utilized the methionine-choline-deficient (MCD) diet to imitate the progression of NAFLD, as first proposed by Shinozuka. In this model, steatohepatitis develops rapidly, with apparent steatohepatitis lesions at about three weeks, liver fibrosis at eight weeks, and significant weight loss [17]. The pathological lesions caused by MCD diet are similar to those of human NAFLD. In addition, we investigated the effects of the GJLZ decoction on lean NAFLD through untargeted metabolomics. The potential metabolic biomarkers related to lean NAFLD were identified, and the underlying mechanism was explored. Our findings might also guide the optimization of TCM treatment for lean NAFLD.

## 2. Results

### 2.1. The Analysis of GJLZ Compositions

**Figure 1:**
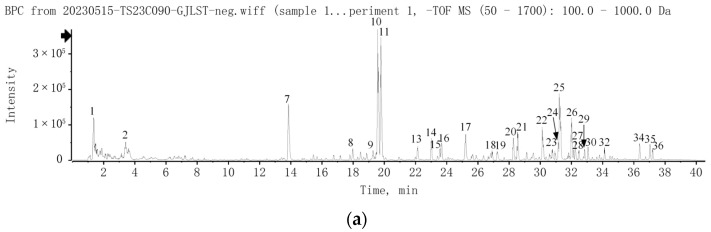

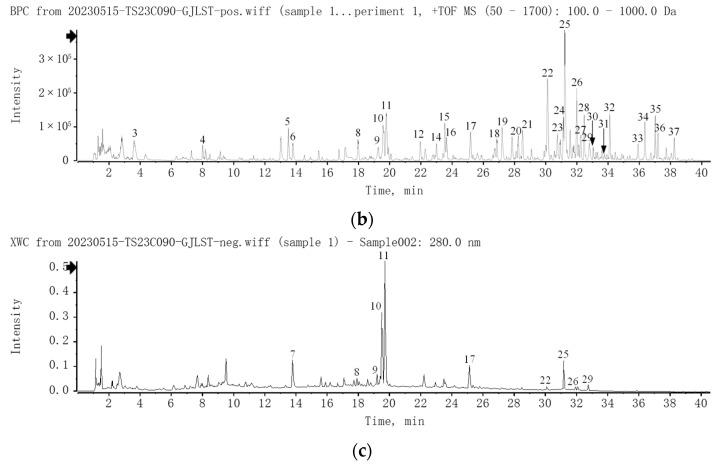
UPLC-Q-TOF/MS analysis of GJLZ decoction. (**a**) The base peak chromatogram of GJLZ decoction via UPLC-HRMS in negative ion mode. (**b**) The base peak chromatogram of GJLZ decoction via UPLC-HRMS in positive ion mode. (**c**) The UV chromatogram of GJLZ decoction at 280 nm. Each number of the peak was identified as the ingredient of GJLZ, the detail information is summarized in Table S3. GJLZ, Ganjianglingzhu.

A total of 37 compounds of GJLZ were identified by UPLC-Q-TOF/MS. The chromatograms are exhibited in Figure 1, and the detailed information about the compounds is summarized in Table S3.

### 2.2. Evaluation of the Lean NAFLD Mode

#### 2.2.1. Body Weight of Mice

**Figure 2:**
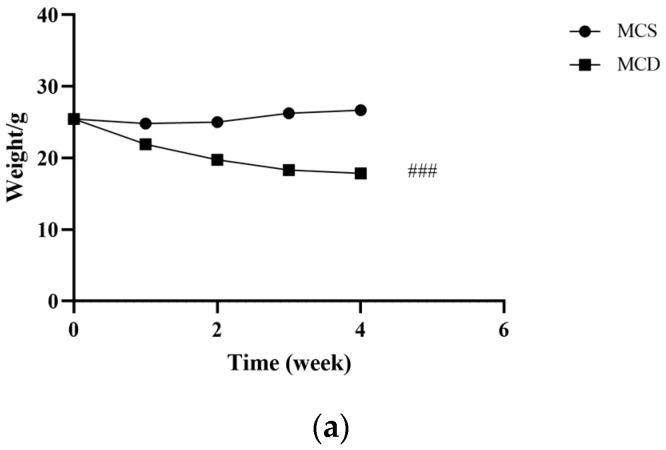

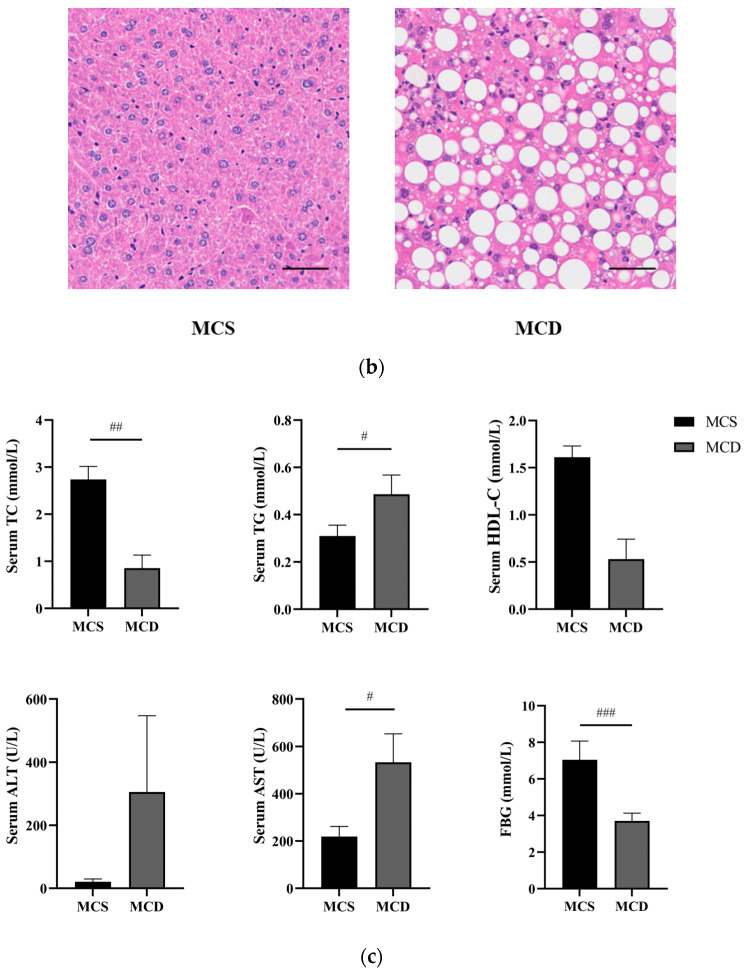
Evaluation of the lean NAFLD model in the fourth week. (**a**) Weight changes in the fourth week. (**b**) H&E staining of hepatic tissue (magnification 200×, the scale bar refers to 50 μm). (**c**) The levels of serum TC, TG, HDL-C, ALT, AST, and FBG. ALT, alanine transaminase; AST, aspartate transaminase; FBG, fasting blood glucose; HDL-C, high-density lipoprotein cholesterol; MCD, methionine-choline-deficient; MCS, methionine-choline-sufficient; NAFLD, non-alcoholic fatty liver disease; TC, total cholesterol; TG, triglyceride. # *p* < 0.050, ## *p* < 0.010 and ### *p* < 0.001: MCD group compared with the MCS group.

After one week of adaptive feeding, no significant difference was observed in body weight between the MCS and the MCD groups. From the first week to the fourth week, the body weight significantly reduced in the MCD group compared with the MCS group, as shown in Figure 2a.

#### 2.2.2. Liver Histology

Morphologic evaluations provided visual evidence of injury from MCD diet-induced NAFLD (Figure 2b). The H&E results showed typical hepatocytes with well-preserved cytoplasm, obvious nucleus and nucleolus, liver lobules, and no steatosis in the MCS group. In the MCD group, we observed the hepatocyte’s steatosis and inflammatory cell infiltration.

#### 2.2.3. Changes in Serum Index of Mice

Compared with the MCS group, serum TG and AST (*p* < 0.05) were significantly increased, and serum TC (*p* < 0.01) and FBG (*p* < 0.001) were significantly decreased, in the MCD group. Serum HDL-C was decreased and serum ALT was increased mainly in the MCD group, although no significant difference was exhibited (Figure 2c). Our results demonstrated that the MCD diet could increase levels of serum liver enzymes, contributing to dyslipidemia and impairing both liver function and lipid metabolism. Based on the abovementioned results, the lean NAFLD model succeeded after four weeks.

### 2.3. Effects of GJLZ on Body Weight

**Figure 3:**
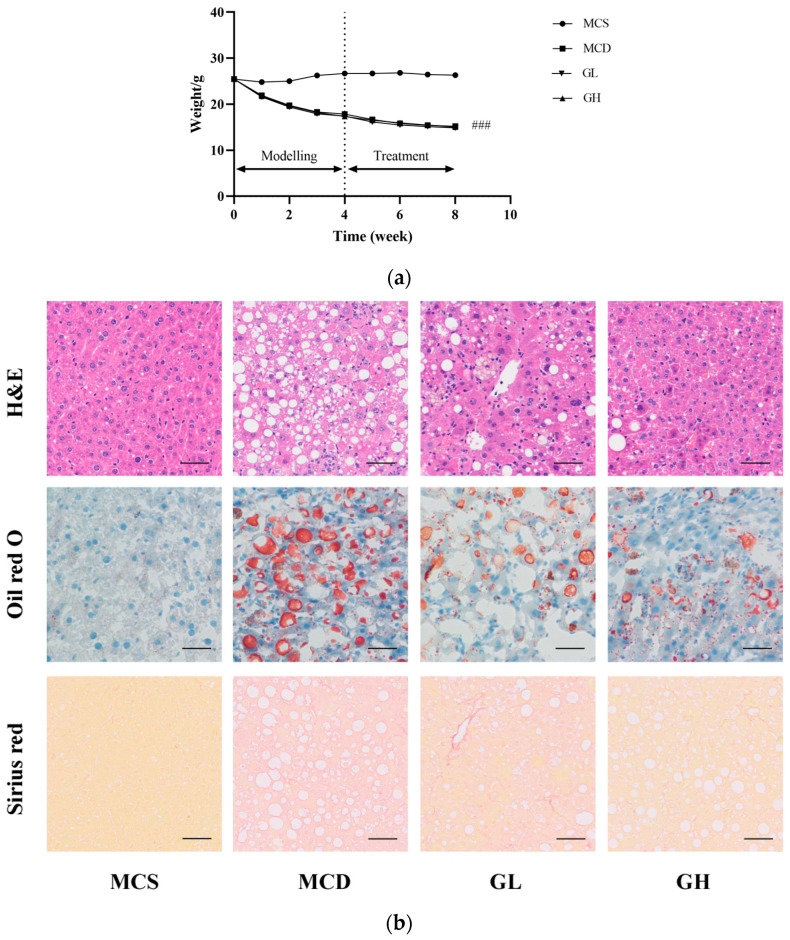

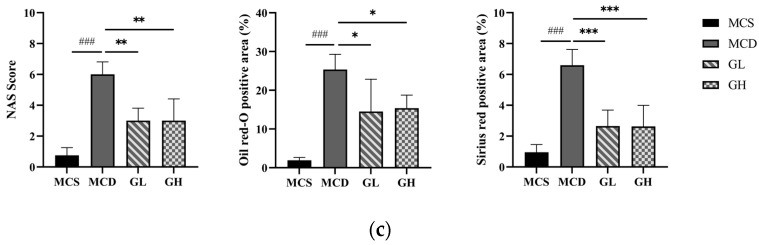
GJLZ ameliorated lean NAFLD induced by the MCD diet. (**a**) Weight changes in the four groups. (**b**) Representative images of hepatic H&E staining, Oil Red O staining, and Sirius Red staining (magnification 200×, the scale bar refers to 50 μm). (**c**) NAS score of liver and histomorphometric analysis of the positive area with Oil Red O staining and Sirius Red staining. GH, high dose of GJLZ; GJLZ, Ganjianglingzhu; GL, low dose of GJLZ; MCD, methionine-choline-deficient; MCS, methionine-choline-sufficient; NAFLD, non-alcoholic fatty liver disease; NAS, NAFLD activity score. ### *p* < 0.001: MCD group compared with the MCS group; * *p* < 0.050, ** *p* < 0.010 and *** *p* < 0.001: GL group and GH group compared with the MCD group.

After eight weeks of the experiment, compared with the MCS group, the body weight (*p* < 0.001) was significantly reduced in the MCD group. Nevertheless, there was no significant difference among MCD, GL, and GH groups. During the treatment, MCD, GL, and GH groups all displayed the trend of weight loss, as shown in Figure 3a.

### 2.4. Effects of GJLZ on Liver Morphology

The liver morphological results revealed severe structural destruction of liver tissue in the MCD group, heavy fat infiltration, hepatomegaly, and inflammatory cell infiltration and fibrosis (Figure 3b). The liver morphology and structure were relatively complete; hepatocellular ballooning was ameliorated, with less inflammatory cell infiltration and fibrosis in the GL and GH groups compared with the MCD group (Figure 3b,c). According to the hepatic histological changes, GL and GH could also significantly decrease NAFLD activity score (NAS) (Figure 3c).

### 2.5. Effects of GJLZ on Serum Index

**Figure 4:**
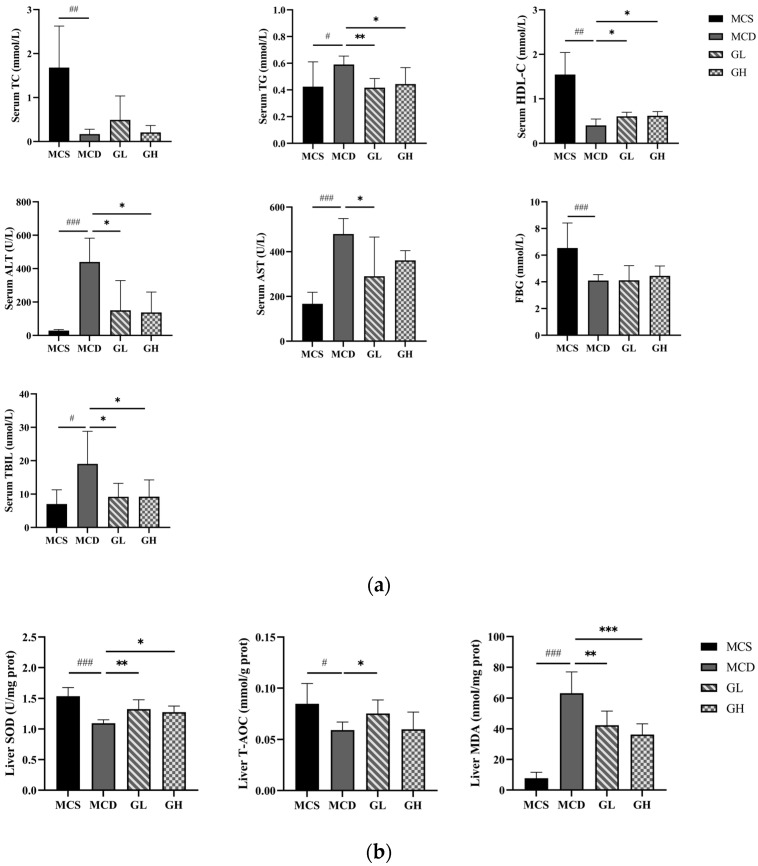
Serum index and liver oxidative stress index among the four groups. (**a**) The levels of serum TC, TG, HDL-C, ALT, AST, FBG, and TBIL. (**b**) The levels of liver SOD, T-AOC, and MDA. ALT, alanine transaminase; AST, aspartate transaminase; FBG, fasting blood glucose; GH, high dose of GJLZ; GJLZ, Ganjianglingzhu; GL, low dose of GJLZ; HDL-C, high-density lipoprotein cholesterol; MCD, methionine-choline-deficient; MCS, methionine-choline-sufficient; MDA, malondialdehyde; SOD, superoxide dismutase; TBIL, total bilirubin; TC, total cholesterol; TG, triglyceride; T-AOC, total antioxidant capacity. # *p* < 0.050, ## *p* < 0.010 and ### *p* < 0.001: MCD group compared with the MCS group; * *p* < 0.050, ** *p* < 0.010 and *** *p* < 0.001: GL group and GH group compared with the MCD group.

Compared with the MCS group, serum TG (*p* < 0.05), ALT, AST (*p* < 0.001), and TBIL (*p* < 0.05) were significantly increased, and serum TC, HDL-C (*p* < 0.01), and FBG (*p* < 0.001) were significantly decreased, in the MCD group. Serum TG (*p* < 0.01), ALT, AST, and TBIL (*p* < 0.05) were significantly decreased, and serum HDL-C (*p* < 0.05) was significantly increased, in the GL group; serum TG, ALT, and TBIL (*p* < 0.05) were significantly decreased, and serum HDL-C (*p* < 0.05) was increased, in the GH group, compared with the MCD group (Figure 4a).

### 2.6. Antioxidative Effects of GJLZ

Previous studies have shown that oxidative stress is of great importance in lean NAFLD. The liver SOD (*p* < 0.001) and T-AOC (*p* < 0.05) were significantly decreased, and the liver MDA (*p* < 0.001) was significantly increased in the MCD group, compared with the MCS group (Figure 4b). The liver SOD and T-AOC were higher, while the liver MDA was lower, in the GL and GH groups than those in the MCD group (SOD, *p* < 0.01; T-AOC, *p* < 0.05; MDA, *p* < 0.01 in the GL group; SOD, *p* < 0.05; MDA, *p* < 0.001 in the GH group, Figure 4b). Overall, the results manifested that GJLZ had the effect of anti-oxidation and alleviating lean NAFLD symptoms.

### 2.7. Untargeted Metabolomics Analysis

#### 2.7.1. Quality Control 

**Figure 5:**
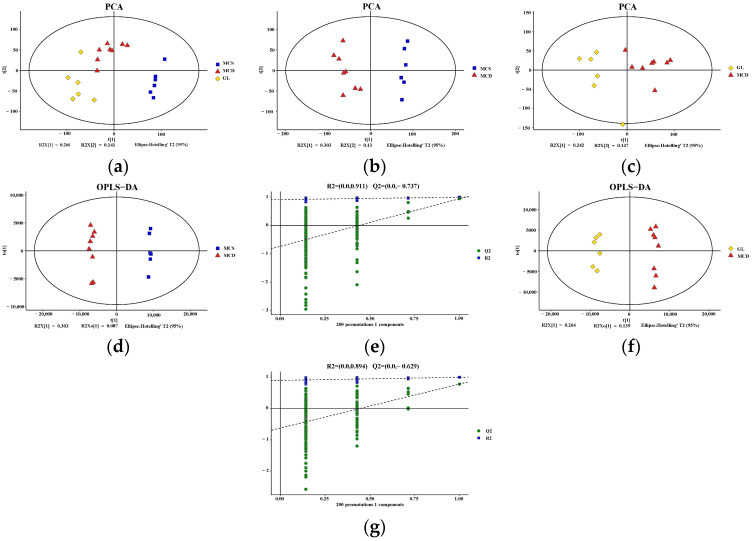
Quality control of the metabolomics data. (**a**) PCA score plot among the three groups. PCA showed a clear group separation between the three groups. (**b**) PCA score plot shows the metabolic state difference between the MCS group and the MCD group. (**c**) PCA score plot showing the difference in the metabolic state between the GL group and MCD group. (**d**,**e**) Score plots of OPLS-DA between the MCS group and the MCD model group and the corresponding coefficient of loading plots. The OPLS-DA models indicated significant metabolic variations between the MCS group and the MCD group. (**f**,**g**) Score plots of OPLS-DA between the GL group and the MCD group and the corresponding coefficient of loading plots. The OPLS-DA models indicated significant metabolic variations between the GL group and the MCD group. GJLZ, Ganjianglingzhu; GL, low dose of GJLZ; MCD, methionine-choline-deficient; MCS, methionine-choline-sufficient; OPLS-DA, orthogonal partial least-squares-discriminant analysis; PCA, principal component analysis.

PCA plots demonstrated a significant separation of liver metabolites between the MCS and MCD groups, as well as a significant separation between the MCD and GL groups (Figure 5a–c). Based on the established OPLS-DA model, the predictive index of the model (Q2) was assessed using seven-fold cross-validation and 200 RPT. Significant segregation of liver metabolites was observed between the MCS group and the MCD group (Figure 5d), as well as between the MCD group and the GL group (Figure 5f), in OPLS-DA models. All samples were within the 95% confidence interval. In the OPLS-DA model, the R2 and Q2 values were (0.0, 0.911) and (0.0, −0.737) between the MCS group and MCD group (Figure 5e), while the R2 and Q2 values were (0.0, 0.894) and (0.0, −0.629) between the MCD group and GL group (Figure 5g).

#### 2.7.2. Cluster Analysis

**Figure 6:**
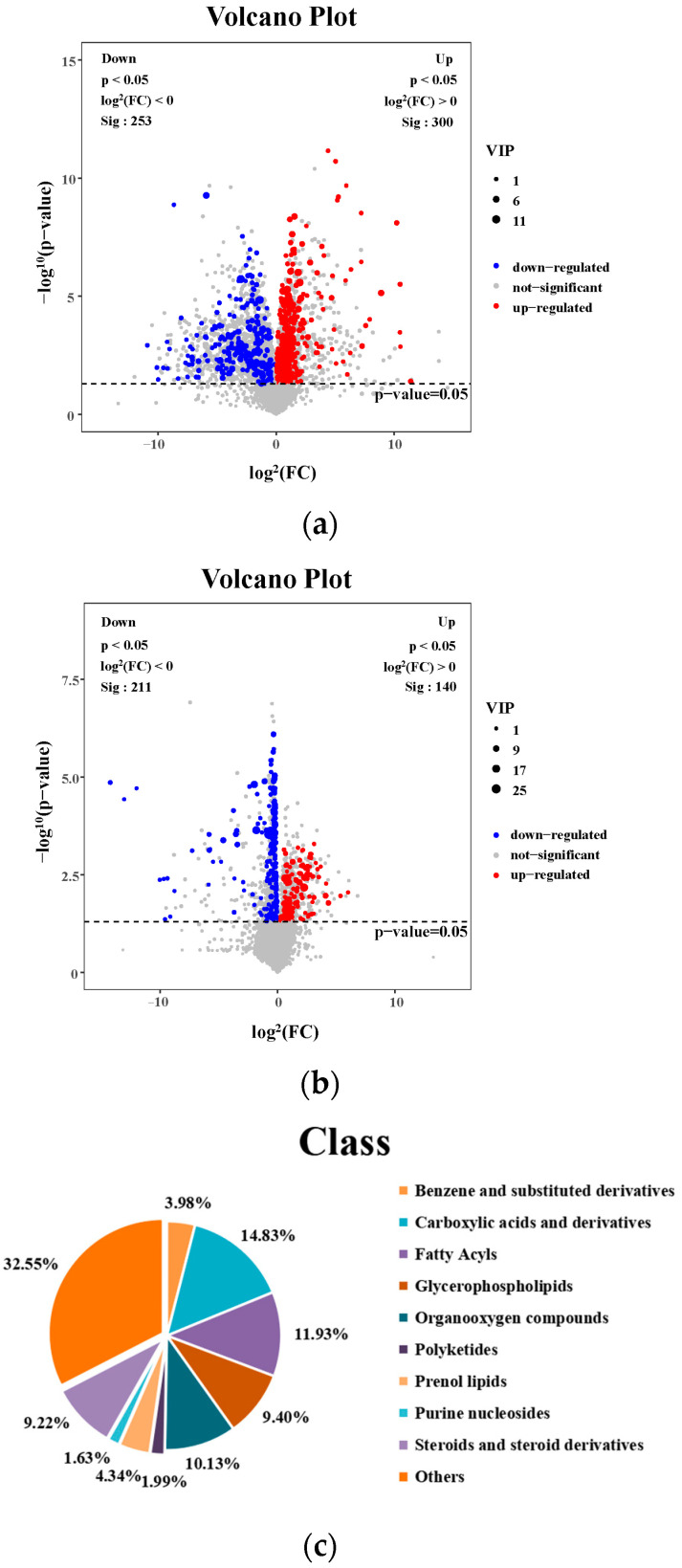

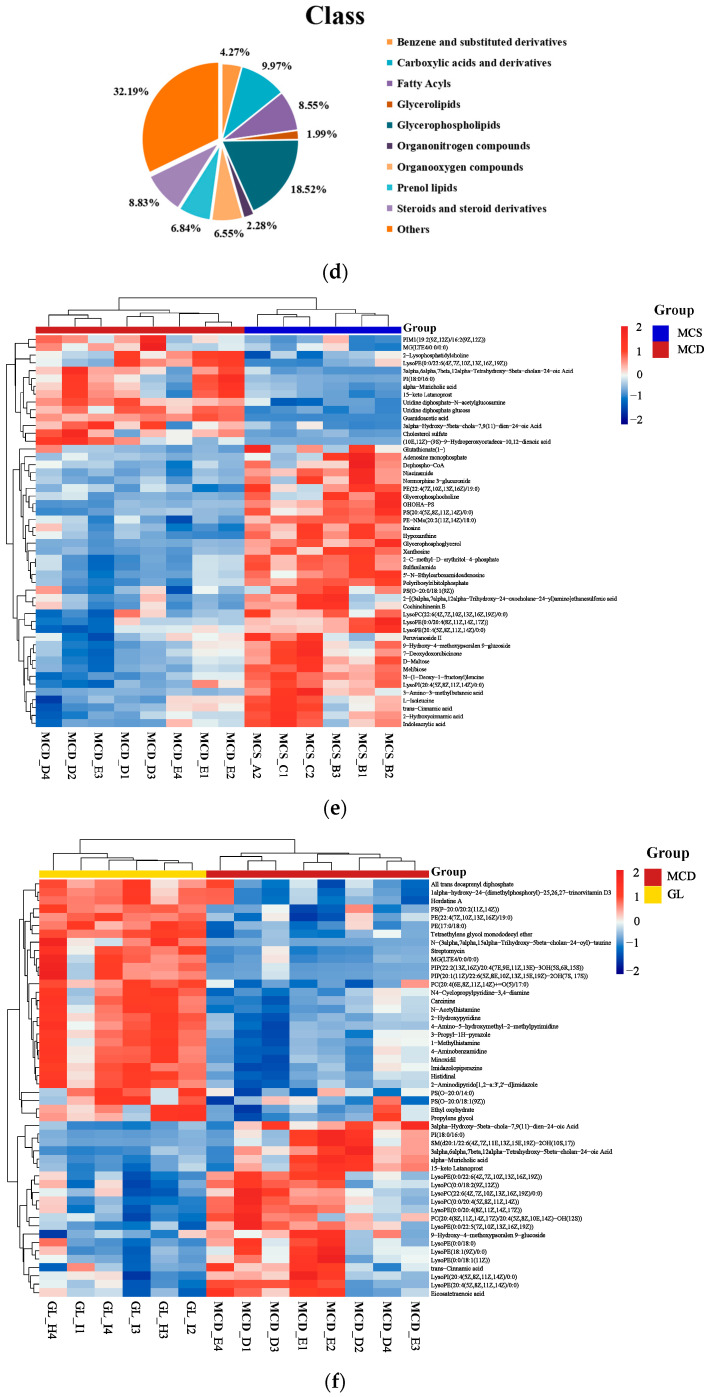
Analysis of different metabolites among three groups. (**a**) Volcano plot for differential metabolites in MCS vs. MCD. (**b**) Volcano plot for differential metabolites in GL vs. MCD. (**c**) Classification of differential metabolites between MCS and MCD groups. (**d**) Classification of differential metabolites between MCD and GL groups. (**e**) Hierarchical cluster analysis heat map of top 50 liver content metabolites in MCS vs. MCD. (**f**) Hierarchical cluster analysis heat map of top 50 liver content metabolites in GL vs. MCD. GJLZ, Ganjianglingzhu; GL, low dose of GJLZ; MCD, methionine-choline-deficient; MCS, methionine-choline-sufficient; VIP, variable importance of projection.

Differential metabolite analysis was performed between the MCS and the MCD groups (VIP > 1 and *p* < 0.05), and a total of 553 liver differential metabolites were identified (Figure 6a); a total of 351 liver differential metabolites were detected between the MCD and the GL groups (VIP > 1 and *p* < 0.05) (Figure 6b). Figure 6c shows that fatty acyls, glycerophospholipids, prenol lipids, steroids, and steroid derivatives were the differential metabolites between MCS and MCD groups, indicating the successful NAFLD modeling. The cluster analysis of the metabolites between MCD and GL groups showed that 44.73% of metabolites were related to fatty acyls, glycerolipids, glycerophospholipids, prenol lipids, steroids, and steroid derivatives (Figure 6d). To more intuitively represent the differences in metabolites, cluster analysis heatmaps were used to classify the top 50 liver differential metabolites between MCS and MCD groups, as well as the principal 50 liver differential metabolites between MCD and GL groups (Figure 6e,f). We selected 78 metabolites that kept the same trends between the MCS and GL groups after comparing them with the MCD group (Table 1 and Table S4).

#### 2.7.3. Metabolite Pathway Enrichment Analysis 

**Figure 7:**
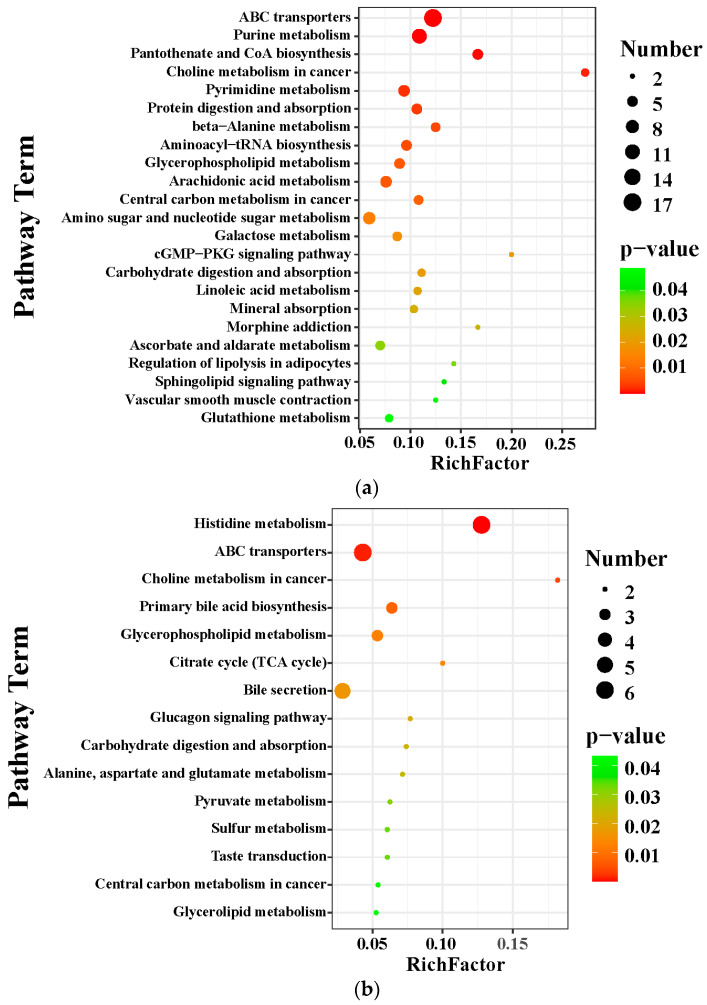

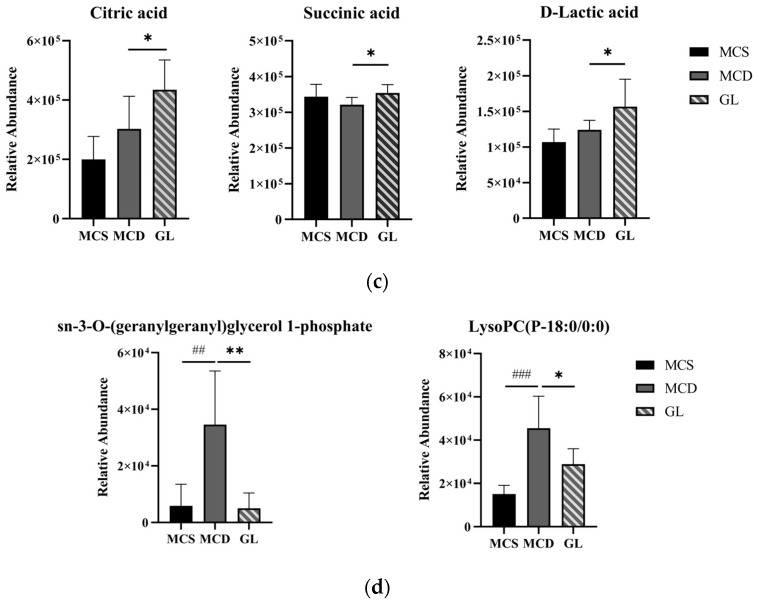
Metabolite pathway enrichment analysis. (**a**) KEGG pathway enrichment analysis between the MCS group and the MCD group. (**b**) KEGG pathway enrichment analysis between the GL group and the MCD group. (**c**) After treatment with GL, the specific differential metabolites in the tricarboxylic acid cycle, glucagon signaling pathway, alanine, aspartate and glutamate metabolism, and pyruvate metabolism. (**d**) In the glycerophospholipid metabolism pathway, sn-3-O-(geranylgeranyl)glycerol 1-phosphate and LysoPC(P-18:0/0:0) were all higher in the MCD group than in the MCS and GL groups. GJLZ, Ganjianglingzhu; GL, low dose of GJLZ; MCD, methionine-choline-deficient; MCS, methionine-choline-sufficient. ## *p* < 0.010 and ### *p* < 0.001: MCD group compared with the MCS group; * *p* < 0.050, and ** *p* < 0.01: GL group compared with the MCD group.

The KEGG database was applied to explore the relevant metabolic pathway of differential metabolites between the MCS and the MCD groups and between the MCD and the GL groups. The analysis of differential metabolic pathways showed significantly affected pathways (*p* < 0.05) and the number of metabolites. When lean NAFLD mice were fed a MCD diet, the perturbed metabolites were primarily concerned with ABC transporters, purine metabolism, pantothenate and CoA biosynthesis, choline metabolism in cancer, pyrimidine metabolism, protein digestion and absorption, beta-alanine metabolism, aminoacyl-tRNA biosynthesis, glycerophospholipid metabolism, arachidonic acid metabolism, central carbon metabolism in cancer, amino sugar and nucleotide sugar metabolism, galactose metabolism, cGMP-PKG signaling pathway, carbohydrate digestion and absorption, linoleic acid metabolism, mineral absorption, morphine addiction, ascorbate and aldarate metabolism, regulation of lipolysis in adipocytes, sphingolipid signaling pathway, vascular smooth muscle contraction, and glutathione metabolism (Figure 7a). After treatment with GL (Figure 7b), the metabolites were related to histidine metabolism, ABC transporters, choline metabolism in cancer, primary bile acid biosynthesis, glycerophospholipid metabolism, citrate cycle (tricarboxylic acid cycle, TCA cycle), bile secretion, glucagon signaling pathway, carbohydrate digestion and absorption, alanine, aspartate and glutamate metabolism, pyruvate metabolism, sulfur metabolism, taste transduction, central carbon metabolism in cancer, and glycerolipid metabolism. We found that citric acid and succinic acid, belonging to the TCA cycle, glucagon signaling pathway, alanine, aspartate, and glutamate metabolism, were significantly increased in the GL group; D-Lactic acid was also elevated in the GL group, which belongs to pyruvate metabolism (Figure 7c). In the glycerophospholipid metabolism pathway, sn-3-O-(geranylgeranyl)glycerol 1-phosphate and lysoPC(P-18:0/0:0) were all higher in the MCD group than in the MCS and GL groups (Figure 7d). Therefore, we focused on the change in sn-3-O-(geranylgeranyl)glycerol 1-phosphate and lysoPC(P-18:0/0:0) in three groups.

### 2.8. Spearman Correlation Analysis of Serum Biochemical Indicators, Liver Antioxidant Indicators, and Differential Metabolites

The Spearman correlation analysis was conducted between serum biochemical indicators, liver antioxidant indicators, and the 78 metabolites selected above (Table S5). Through this analysis, we discovered that two metabolites belonging to the glycerophospholipid metabolism pathway were closely associated with these indicators. Sn-3-O-(geranylgeranyl)glycerol 1-phosphate was positively correlated with serum TG, AST, and liver MDA, while it was negatively correlated with liver SOD; lysoPC(P-18:0/0:0) was positively correlated with serum ALT, AST, and liver MDA, while it was also negatively correlated with serum HDL-C, liver SOD, and T-AOC (Table 2).

## 3. Discussion

Lean NAFLD patients exhibit a normal BMI but have insulin resistance, excessive visceral adiposity, and metabolic dysfunction, which are typically observed in obese individuals [18]. At present, there is an increasing consensus that lean NAFLD may be associated with genetic and environmental factors [19,20,21]. At the same time, there is no proven medical treatment for lean NAFLD [22]. Therefore, we designed an experiment to verify whether GJLZ is effective in treating lean NAFLD. The results showed the following: (1) GJLZ can alleviate the degree of steatosis, inflammation, fibrosis, and oxidative stress injury in the liver and antagonize the accumulation of lipids in serum. (2) GJLZ improves glucose and lipid metabolism in lean NAFLD. (3) GJLZ can regulate glycerophospholipid metabolism, and the two metabolites from the glycerophospholipid metabolism pathway: sn-3-O-(geranylgeranyl)glycerol 1-phosphate and lysoPC(P-18:0/0:0) might become novel targets for lean NAFLD.

In this research, we measured body weight and serum biochemical parameters and evaluated liver morphology to trial the effect of the MCD diet in modeling lean NAFLD and the effectiveness of GJLZ in treating lean NAFLD. The MCD diet was able to reduce weight, FBG, serum TC, and HDL-C; elevate serum TG, ALT, AST, and TBIL; and cause steatosis, inflammation lobular, hepatocellular ballooning, and fibrosis in the liver, which was consistent with the results of previous research [23]. After treatment with GJLZ, we found different doses of GJLZ ameliorate steatosis, inflammation, and fibrosis in the liver, lessen serum lipid accumulation, and reduce liver damage. This is consistent with the research of Dang et al., which showed that GJLZ decoction improved liver steatosis and inflammation, alleviated the liver injury, and considerably reduced serum AST and ALT contents in NAFLD [15].

Oxidative stress in the liver is important in the progression of lean NAFLD. SOD, T-AOC, and MDA are the leading indices that measure the antioxidant capacity. Liver MDA is the lipid peroxidation product that was elevated in the MCD group when compared with the MCS, GL, and GH groups; SOD, which is an antioxidant enzyme that makes significant contributions to diminish oxidative stress, was lower in the MCD group when compared with MCS, GL, and GH groups [24,25]. The liver T-AOC level was lower in the MCD group than in the MCS and GL groups. The MCD diet increased liver oxidative stress and decreased the antioxidant capacity, exacerbating lean NAFLD. GL and GH reduced the MDA level and improved the liver SOD level; GL also raised liver T-AOC. Those findings illustrate that different doses of GJLZ improved liver oxidative stress by enhancing the content of SOD and suppressing the production of MDA; GJLZ could be a prophylactic medicine in lean NAFLD. The same results for Poria cocos polysaccharides in improving liver T-AOC and reducing liver MDA levels, one of the components in Poria from GJLZ, support our research [26]. 

To further explore the therapeutic mechanism of GJLZ in lean NAFLD, we chose GL as a better dosage for treating lean NAFLD according to the careful consideration of serum biochemicals, hepatic oxidative stress level, and histological analysis. Based on metabolomics analysis, we discovered an abundance of differential metabolic pathways between MCS and MCD groups; glycerophospholipid metabolism, arachidonic acid metabolism, galactose metabolism, carbohydrate digestion and absorption, linoleic acid metabolism, regulation of lipolysis in adipocytes, and sphingolipid signaling pathway were related to glucose and lipid metabolism, indicating that MCD diets not only cause liver lipid accumulation, but also induce glucose and lipid metabolism disorder. This is in accordance with the research of Ye et al. on metabolic disorders in lean NAFLD, which showed that metabolic diseases such as hypercholesterolemia, diabetes, and elevated blood pressure, or both elevated blood pressure and hypercholesterolemia, should be the screening criteria for lean NAFLD, besides BMI [27]. After GL treatment, the pathways related to glucose and lipid metabolism: glycerophospholipid metabolism, glucagon signaling pathway, carbohydrate digestion and absorption, and glycerolipid metabolism were altered. Glucagon can stimulate lipolysis in adipocytes. The activation of the glucagon signaling pathway may illustrate that GL can enhance the lipolysis of the adipocytes in the liver to alleviate lean NAFLD [28,29]. The accumulation of hepatocyte lipids in NAFLD triggers the production of reactive oxygen species and the rise in oxidative stress [30]. These processes ultimately lead to mitochondrial dysfunction, which may cause the abnormal process of the TCA cycle. Research shows that decreased glycolytic/TCA cycle metabolites are associated with cirrhosis; we can infer that the down-regulation of glycolytic/TCA cycle metabolites may be linked to liver injury [29]. In the fasted state, lactate and amino acids fail to produce glucose via gluconeogenesis. TCA cycle intermediates accumulate in hepatocytes, leading to hepatic steatosis [31]. The up-regulation of the TCA cycle and pyruvate metabolism pathway and their metabolites suggests that GL may improve glucose and lipid metabolism in lean NAFLD.

Lipids are a group of complex and diverse molecules; lipid alterations are a common cause and consequence of NAFLD, alcoholic hepatitis, and steatohepatitis [32]. Glycerophospholipids are involved in cellular signal transduction, the major component of cellular membranes. The phospholipid family is classified into phosphatidic acid (PA), phosphatidylcholine (PC), phosphatidylethanolamine (PE), phosphatidylinositol (PI), phosphatidylserine (PS), cardiolipin (CL), etc. [33]. Glycerophospholipid metabolism involves diseases like type 2 diabetes mellitus and atherosclerosis, psoriasis, and tuberculosis comorbidity [34,35,36]. When analyzing the 78 common metabolites in three groups, we focused on two metabolites in glycerophospholipid metabolism: sn-3-O-(geranylgeranyl)glycerol 1-phosphate and lysoPC(P-18:0/0:0), which decreased in the MCS and GL groups and increased in the MCD group. LysoPC has diverse roles in cellular processes, including regulating inflammation, cell differentiation, immune responses, and signaling pathways in various cell types [37,38,39]. The elevated lysoPC(15:0) content was causally related to the risk of high uric acid, high insulin, high homeostasis model assessment of insulin resistance (HOMA-IR), dyslipidemia, and overweight/obesity, which shows that abnormal lysoPC metabolism may be connected to the metabolic risk [33,40]. Likewise, the dynamic changes in sn-3-O-(geranylgeranyl)glycerol 1-phosphate and lysoPC(P-18:0/0:0) may reflect the injury and recovery of the liver. This suggests that the GL treatment is effective in lean NAFLD, probably by regulating the glycerophospholipid metabolism. Moreover, sn-3-O-(geranylgeranyl)glycerol 1-phosphate and lysoPC(P-18:0/0:0) may serve as biomarkers for lean NAFLD and have a negative effect on lean NAFLD. 

**Figure 8:**
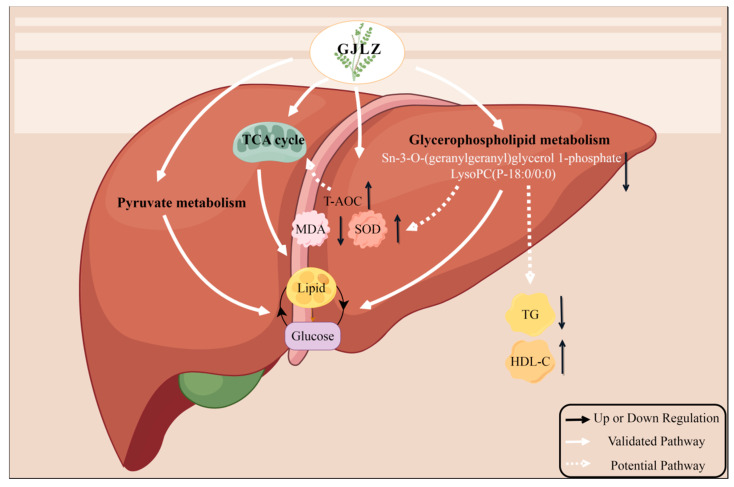
The mechanisms underlying the therapeutic effects of GJLZ in treating lean NAFLD. GJLZ, Ganjianglingzhu; HDL-C, high-density lipoprotein cholesterol; MDA, malondialdehyde; NALFD, non-alcoholic fatty liver disease; SOD, superoxide dismutase; TCA cycle, tricarboxylic acid cycle; TG, triglyceride; T-AOC, total antioxidant capacity. The cartoon components originated from www.figdraw.com for model drawing (accessed on 28 November 2023).

To provide more evidence that the level of sn-3-O-(geranylgeranyl)glycerol 1-phosphate and lysoPC(P-18:0/0:0) may be associated with the severity of lean NAFLD, we conducted a correlation analysis of serum biochemical indicators and liver antioxidant indicators, sn-3-O-(geranylgeranyl)glycerol 1-phosphate and lysoPC(P-18:0/0:0). They were positively correlated with serum ALT and AST, indicating sn-3-O-(geranylgeranyl)glycerol 1-phosphate and lysoPC(P-18:0/0:0) may be involved in liver damage. They were positively correlated with serum TG, while being negatively correlated with serum HDL-C; this shows that sn-3-O-(geranylgeranyl)glycerol 1-phosphate and lysoPC(P-18:0/0:0) could lead to dyslipidemia in lean NAFLD. This is consistent with the outcomes of Han et al., which showed that patients with coexisting HBV infection, NAFLD, and T2DM exhibit higher levels of sn-3-o-(geranylgeranyl) glycerol1-phosphate and serum TG, which may infer that the increase in sn-3-o-(geranylgeranyl) glycerol1-phosphat indicates the excessive lipogenesis [41]. Sn-3-O-(geranylgeranyl)glycerol, 1-phosphate, and lysoPC(P-18:0/0:0) were positively associated with MDA and inversely associated with T-AOC and SOD, suggesting that sn-3-O-(geranylgeranyl)glycerol, 1-phosphate, and lysoPC(P-18:0/0:0) may exacerbate oxidative stress in the liver. Our discovery is in accordance with the study of Pang et al., which showed that the HFD diet induced NAFLD with higher HDL, LDL, TC, TG, FBG, ALT, and AST levels that were positively correlated with the PC and PE levels [42]. Furthermore, Zhou et al. reported that metabolism of glycerophospholipid was highly associated with the hepatic injury triggered by Concanavalin A (Con A), which showed that the glycerophospholipid metabolism pathway was of great importance in Con A-induced hepatic injury [43]. The correlation analysis of serum biochemical indicators, liver antioxidant indicators, sn-3-O-(geranylgeranyl)glycerol 1-phosphate, and lysoPC(P-18:0/0:0) demonstrated that GL may regulate glycerophospholipid metabolism by reducing sn-3-O-(geranylgeranyl)glycerol 1-phosphate and lysoPC(P-18:0/0:0) levels, thereby ameliorating glucose and lipid metabolism disorder and oxidative stress in liver, as well as hepatic injury. In summary, treating GJLZ could enhance the antioxidant capacity of liver cells, recover the TCA cycle function, and improve pyruvate and glycerophospholipid metabolism. Restoration of glycerophospholipid metabolism leads to the downregulation of sn-3-O-(geranylgeranyl)glycerol 1-phosphate and lysoPC(P-18:0/0:0) levels. It is associated with the elevation of serum HDL-C, hepatic T-AOC, and SOD, and the reduction in serum TG and hepatic MDA, thereby restoring glucose and lipid metabolism (Figure 8).

We made several discoveries in this study. Firstly, we found that GJLZ, an effective medication from TCM, may treat lean NAFLD. Secondly, according to untargeted metabolomics analysis, we found that GL ameliorated lean NAFLD by regulating glucose and lipid metabolism, and sn-3-O-(geranylgeranyl)glycerol 1-phosphate and lysoPC(P-18:0/0:0) were screened from the glycerophospholipid metabolism pathway. However, this research has some limitations. First, the number of mice was six to eight in each group; therefore, a larger sample size is required to verify the accuracy of our experimental outcomes. Second, there are no general methods to model lean NAFLD in mice. Thus, the MCD diet mouse model was constructed to model lean NAFLD, which significantly impacted the mouse’s longevity and failed to demonstrate metabolic features, including the usual insulin resistance and visceral fat accumulation, in lean NAFLD patients. Still, it is of interest to study the pathogenesis of lean NAFLD. Third, lipidomics must be applied in subsequent research after we revealed the importance of lipid metabolism in the treatment of lean NAFLD using GJLZ. Fourth, in our research we did not explore the beneficial effects of GJLZ supplementation on lean NAFLD due to direct effects on the liver or due to indirect effects via other tissues; thus, more experiments are needed to further investigate the beneficial effects of GJLZ on lean NAFLD. Finally, genomics, transcriptomics, and proteomics should be utilized in the continuing exploration to systematically search for the mechanism.

## 4. Materials and Methods

### 4.1. Preparation of GJLZ Decoction

The original prescription of the GJLZ decoction comprised Glycyrrhizae Radix et Rhizoma (6 g), Zingiberis Rhizoma (12 g), Poria (12 g), and Macrocephalae Rhizoma (6 g), which was set as the low-dose group of the GJLZ decoction (GL group). The dosage of the high-dose group of the GJLZ decoction (GH group) was 1.5 times that of the GL group. We purchased granules from Sichuan Neo-Green Pharmaceutical Technology Development Co., Ltd. (Chengdu, China). A quantity of 1g of Glycyrrhizae Radix et Rhizoma, Zingiberis Rhizoma, Poria, and Macrocephalae Rhizoma granules was equivalent to 7 g, 21 g, 17 g, and 7 g of herbs, respectively. The granules were mixed according to the proportion of the GJLZ decoction. The dose of intragastric administration in mice was calculated as 12.33 times the clinical adult dose; the low dose (614.70 g·kg^−1^·d^−1^) and high dose (922.05 g·kg^−1^·d^−1^) of the GJLZ decoction were administered to mice in the experiment.

### 4.2. Component Analysis of GJLZ Decoction

The composition of the GJLZ decoction was analyzed by UPLC-Q-TOF/MS. The ultra-high-performance liquid chromatography (UPLC) system was Waters H-Class UPLC system (Waters Corporation, Milford, MA, USA), the chromatographic column was a Waters ACQUITY UPLC^®^ HSS T3 (2.1 × 150 mm, 1.8 µm, Waters Corporation, Milford, MA, USA), and the column temperature was 30 °C. The sample injection volume was 2 μL. The mobile phase was an aqueous solution of acetonitrile (A) and 0.1% formic acid (B); the DAD wavelength ranged from 190 to 400 nm. The flow rate was 0.3 mL/min. The gradient elution program is exhibited in Table S1.

The AB Sciex Triple TOF^®^ 4600 system (AB SCIEX LLC, Framingham, MA, USA) with an ESI ion source was used to perform MS analysis. Samples were analyzed in negative and positive ion modes; the parameter set is shown in Table S2.

### 4.3. Mouse Preparation and Sampling

Male C57BL/6 mice (9-week-old; n = 38) were immediately kept in a specific pathogen-free room after being purchased from the Vital River (Beijing, China). After being adaptively fed for one week and weighed, they were randomly divided into a lean NAFLD model group (provided with the MCD diet, #519580, Dyets, Wuxi, China), having 27 mice, and a control group (provided with the methionine-choline-sufficient (MCS) diet, #519581, Dyets, Wuxi, China), having 11 mice. After feeding of the MCD and MCS diets for four weeks, three mice from the MCD diet model and MCS diet control groups were sacrificed. To estimate the model, serum total cholesterol (TC), triglyceride (TG), high-density lipoprotein cholesterol (HDL-C), alanine transaminase (ALT), aspartate transaminase (AST), and fasting blood glucose (FBG) were determined, and the liver tissues of mice were stained with hematoxylin, eosin (H&E).

Subsequently, the lean NAFLD model group was randomly divided into three groups: model group (MCD group, n = 8), GL group (n = 8, 614.70 g·kg^−1^·d^−1^), and GH group (n = 8, 922.05 g·kg^−1^·d^−1^). The GL group and GH group received intragastric administration of varying concentrations of the GJLZ decoction; the control group (MCS group, n = 8) and MCD group were given 7.5 mL·kg^−1^·d^−1^ physiological saline by gavage. In the eighth week, the mice from each group were sacrificed, and arterial blood and liver tissue were taken and immediately stored at −80 °C. The animal experiments were approved by the Animal Ethics Committee of the Shanghai Model Organisms Center (IACUC NO. 2022-0034-1).

### 4.4. Histological Analysis of Liver Tissue

The liver tissues of mice fixed with paraformaldehyde were made into paraffin sections and stained with H&E and Sirius Red. Frozen sections were utilized for Oil Red O staining. The staining area was calculated by ImageJ-win64.

### 4.5. Serum Biochemical and Liver Oxidative Stress Indicator Analysis

An automatic biochemical analyzer detected the serum TC, TG, HDL-C, ALT, AST, and total bilirubin (TBIL) (Chemray 240, Rayto, Shenzhen, China). The liver malondialdehyde (MDA), superoxide dismutase (SOD), and total antioxidant capacity (T-AOC) were measured using the assay kits from Nanjing Jiancheng Bioengineering Institute according to the instructions (Nanjing, China).

### 4.6. Untargeted Metabolomics Analysis

L-2-chlorophenylalanine was purchased from Shanghai Heng Chuang Bio-technology Co., Ltd. (Shanghai, China). Acetonitrile, formic acid, methanol, and water were acquired from Thermo Fisher Scientific (Thermo Fisher Scientific, Waltham, MA, USA). All reagents were appropriate for the high-performance liquid chromatography analysis. 

#### 4.6.1. Metabolite Extraction 

The liver sample was weighed (30 mg) and then transferred to a 1.5 mL tube with two small steel balls. A mixture of 400 μL of methanol and water (4/1, *v/v*) and 4 μg/mL of L-2-chlorophenylalanine (internal standard) were added to each sample. A quantity of 300 μL of the supernatant was taken after grinding the samples and centrifugation. A further 300 μL of methanol and water (1/4, *v/v*) was added to each sample and the supernatant (150 μL) was then collected in each tube. Aliquots of all samples were pooled to make quality control (QC) samples.

#### 4.6.2. UPLC–MS/MS Analysis 

The metabolic profiling was analyzed using the VION IMS QTOF mass spectrometer (Waters Corporation, Milford, MA USA) and ACQUITY UPLC I-Class system (Waters Corporation, Milford, MA, USA). The chromatographic column was an ACQUITY UPLC HSS^®^ T3 (2.1 × 100 mm, 1.8 µm), and the column temperature was 45 °C. The sample injection volume was 3 μL. The mobile phase was composed of an aqueous solution of A (water) and B (acetonitrile/methanol 2/3 (*v*/*v* 0.1%) plus 0.1% formic acid; the flow rate was 0.4 mL/min. The data were collected using a combination of full scan mode (*m*/*z* ranging from 50 to 1000) and MSE mode, alternating two independent scans during operation with different collision energies. The QCs were utilized to provide the data that could be evaluated for reproducibility by periodically injecting them throughout the analysis.

#### 4.6.3. Bioinformatics Analysis 

The raw LC-MS data were processed by the Proggenesis QI V2.3 software (Nonlinear, Dynamics, Newcastle, UK) for baseline filtering, peak identification, peak extraction, peak alignment, and normalization. Compounds were identified by secondary fragmentation, mass-to-charge ratio, and isotopic distribution. The qualitative analysis was based on the Human Metabolome Database (HMDB), Lipidmaps (V2.3), EMDB, PMDB, METLIN, and self-built databases. The data matrix consisted of a combination of positive and negative ion data. 

Principal component analysis (PCA) was utilized for estimating the analysis process’s stability and the samples’ distribution through R. Differential metabolites between groups were identified by orthogonal partial least-squares-discriminant analysis (OPLS-DA). Seven-fold cross-validation and 200 response permutation testing (RPT) were used to prevent model overfitting. The two-tailed Student’s *t*-test and the variable importance of projection (VIP) values in the OPLS-DA model were used to screen out differential metabolites (VIP > 1.0 and *p* < 0.05). The KEGG database (http://www.kegg.jp) was used to identify the differential biological pathways of metabolite data, which was accessed on 14 September 2023.

### 4.7. Statistical Analysis

SPSS 26.0 software was used for statistical analyses. The two-tailed Student’s *t*-test (between two groups) and one-way analysis of variance (ANOVA) (between multiple groups) were used for the data that had a normal distribution, and the nonparametric test was utilized for non-normal distributions. Data are presented as the mean ± SD in this study.

## 5. Conclusions

The efficacy and mechanism of GJLZ in treating lean NAFLD were studied for the first time, and the low dose (i.e., the original prescription dose) was found to have a better therapeutic effect on lean NAFLD. The analysis of untargeted metabolomics exhibited that GL may regulate glucose and lipid metabolism to alleviate lean NAFLD. Glycerophospholipid metabolism plays a crucial role in the treatment of lean NAFLD using GL. Furthermore, sn-3-O-(geranylgeranyl)glycerol 1-phosphate and lysoPC(P-18:0/0:0) are likely to be involved in aggravating lean NAFLD and may serve as diagnostic and therapeutic biomarkers for lean NAFLD in the future.

## Figures and Tables

**Table 1:** List of the top 10 identified metabolites of the intersection statistics between MCS vs. MCD and MCD vs. GL in positive and negative ion modes based on UHPLC-Q-TOF/MS.

*m*/*z*	Retention Time (min)	Ion Mode	Formula	Metabolites	VIP	*p*-Value	FC	
							MCS vs. MCD	MCD vs. GL
453.2860415	7.848233333	neg	C24H40O5	alpha-Muricholic acid	16.06769146	0.001366585	0.226549791	4.844474413
854.5920444	11.47483333	neg	C46H84NO8P	PE(22:4(7Z,10Z,13Z,16Z)/19:0)	11.85212432	0.000161857	1.861265702	0.653574112
373.2735342	8.718383333	pos	C24H36O3	3alpha-Hydroxy-5beta-chola-7,9(11)-dien-24-oic Acid	10.53291874	0.001324031	0.254796555	4.932638804
469.2808627	6.996783333	neg	C24H40O6	3alpha,6alpha,7beta,12alpha-Tetrahydroxy-5beta-cholan-24-oic Acid	9.722835203	0.00032875	0.108749729	5.89703359
786.6001094	12.6016	pos	C44H86NO9P	PS(O-20:0/18:1(9Z))	9.283616715	0.016021262	1.313988022	0.796299615
526.2928203	10.2723	pos	C27H44NO7P	LysoPE(0:0/22:6(4Z,7Z,10Z,13Z,16Z,19Z))	9.248142084	0.002656101	0.589762419	1.94542687
475.2678339	7.848233333	neg	C26H38O5	15-keto Latanoprost	7.181839741	0.001131022	0.312034456	3.641811108
837.5497715	7.848233333	neg	C43H83O13P	PI(18:0/16:0)	5.834670211	0.002426321	0.074756485	17.15141474
113.0713445	14.8042	pos	C5H5NO	2-Hydroxypyridine	5.509739735	7.52517 × 10^−6^	1.075065689	0.845072861
165.113551	14.23996667	pos	C8H14N4O	Carcinine	5.38750534	0.000454935	1.071885203	0.879479787

FC, fold change; GJLZ, Ganjianglingzhu; GL, low dose of GJLZ; MCD, methionine-choline-deficient; MCS, methionine-choline-sufficient; VIP, variable importance of projection.

**Table 2:** The correlation between biochemical indicators and the metabolites.

	TG	HDL-C	ALT	AST	TBIL	SOD	T-AOC	MDA
sn-3-O-(geranylgeranyl)glycerol 1-phosphate	0.560 *	−0.443	0.475	0.569 *	0.29	−0.624 **	−0.354	0.649 **
LysoPC(P-18:0/0:0)	0.362	−0.896 **	0.741 **	0.800 **	0.293	−0.706 **	−0.737 **	0.738 **

ALT, alanine transaminase; AST, aspartate transaminase; HDL-C, high-density lipoprotein cholesterol; MDA, malondialdehyde; SOD, superoxide dismutase; TBIL, total bilirubin; TG, triglyceride; T-AOC, total antioxidant capacity. * Correlation is significant at the 0.05 level (2-tailed); ** correlation is significant at the 0.01 level (2-tailed).

## Data Availability

The data presented in this study are available on request from the corresponding author.

## Supplementary Materials

The following supporting information can be downloaded at: https://www.mdpi.com/article/10.3390/ph17040502/s1, Table S1: Elution gradient; Table S2: Mass parameters (Sciex Triple TOF 4600 LC-MS); Figure S1: Food intake of mice; Table S3: Main component identification of GJLZ decoction [44,45,46,47,48]; Table S4: List of the identified metabolites of the intersection statistics between MCS vs. MCD and MCD vs. GL in positive and negative ion mode based on UHPLC-Q-TOF/MS; Table S5: The correlation between biochemical indicators and the metabolites.

## Abbreviations

ALT: alanine transaminase; ANOVA: analysis of variance; AST: aspartate transaminase; BMI: body mass index; CL: cardiolipin; Con A: Concanavalin A; FBG: fasting blood glucose; FC: fold change; GH: high dose of GJLZ; GJLZ: Ganjianglingzhu; GL: low dose of GJLZ; HDL-C: high-density lipoprotein cholesterol; HMDB: human metabolome database; HOMA-IR: homeostasis model assessment of insulin resistance; H&E: hematoxylin, eosin; MCD: methionine-choline-deficient; MCS: methionine-choline-sufficient; MDA: malondialdehyde; NAFLD: non-alcoholic fatty liver disease; NAS: non-alcoholic fatty liver disease activity score; NASH: non-alcoholic steatohepatitis; OPLS-DA: orthogonal partial least-squares-discriminant analysis; PA: phosphatidic acid; PC: phosphatidylcholine; PCA: principal component analysis; PE: phosphatidylethanolamine; PI: phosphatidylinositol; PS: phosphatidylserine; QC: quality control; RPT: response permutation testing; SOD: superoxide dismutase; TBIL: total bilirubin; TC: total cholesterol; TCA cycle: tricarboxylic acid cycle; TCM: traditional Chinese medicine; TG: triglyceride; T-AOC: total antioxidant capacity; UPLC: ultra-high-performance liquid chromatography; VIP: variable importance of projection.
