# Supplementary material for: Effects of Ganjianglingzhu Decoction on Lean Non-Alcoholic Fatty Liver Disease in Mice Based on Untargeted Metabolomics
Source: Pharmaceuticals (Basel). 2024 Apr 15;17(4):502. doi: 10.3390/ph17040502 (PMC11053674; doi:10.3390/ph17040502)
Supplement: Supplementary file 1 [file pharmaceuticals-17-00502-s001.zip › pharmaceuticals-2886773-supplementary.pdf]

Table S1. Elution gradient

Time (min)	A%	B%
0~3	0	100
3~7	0~7	100~93
7~10	7~10	93~90
10~28	10~40	90~60
28~38	40~95	60~5
38~42	95	5
42~42.1	95~0	5~100
42.1~45	0	100

Table S1. Mass parameters (Sciex Triple TOF 4600 LC-MS)

MS parameters	Values	MS/MS parameters	Values
TOF mass range	50~1700	MS/MS mass range	50~1250
Ion Source Gas 1（psi）	50	Declustering Potential（V）	100
Ion Source Gas 2（psi）	50	Collision Energy（eV）	±40
Curtain Gas（psi）	35	Collision Energy Spread（eV）	20
Ion Spray Voltage Floating (V)	-4500/5000	Ion Release Delay（ms）	30
Ion Source Temperature (°C)	500	Ion Release Width（ms）	15
Declustering Potential（V）	100		
Collision Energy（eV）	10		

Figure S1. Food intake of mice

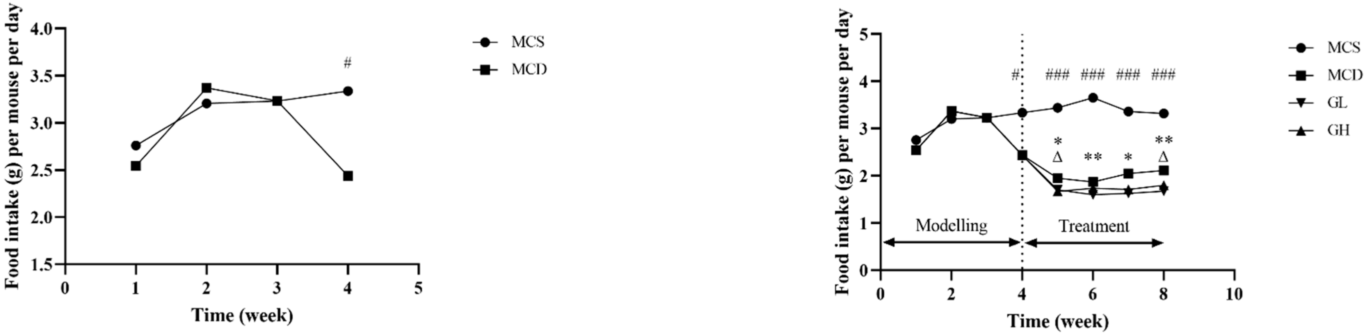

GH: high dose of GJLZ; GJLZ: Ganjianglingzhu; GL: low dose of GJLZ; MCD: methio-nine-choline-deficient; MCS: methionine-choline-sufficient.

#p < 0.050, and ###p < 0.001: MCD group compared with the MCS group; *p < 0.050, and **p < 0.010 : GL group compared with the MCD group; Δp < 0.050: GH group compared with the MCD group.

Table S3. Main component identification of GJLZ decoction

No.	RT (min)	Adduct ions	Measured m/z	Respected m/z	ppm	Formula	M.W.	Identification	MS/MS data	Source	CAS Number	Peak Area	Component Percentage	Data-base	Refer-ences
1	1.36	$[M-H]^-$	195.0506	195.0510	-2.2	$C_6H_{12}O_7$	196.06	Gluconic acid	195.0493;129.0189;75.0085;59.0132	Glycyrrhizae Radix et Rhizoma; Zingiberis Rhlzoma	526-95-4	4093177	4.02%		
2	3.41	$[M-H]^-$	191.0195	191.0197	-1.2	$C_6H_8O_7$	192.03	Citric acid	191.0190;111.0072;87.0073;67.0171;57.0330	All	77-92-9	2829467	2.78%	√	
3	3.62	$[M+H]^+$	144.1019	144.1019	0.0	$C_7H_{13}NO_2$	143.09	Stachydrine	144.1021;84.0812;56.0475	Glycyrrhizae Radix et Rhizoma; Zingiberis Rhlzoma	471-87-4	3098821	3.05%	√	
4	8.01	$[M+H]^+$	268.1036	268.1040	-1.6	$C_{10}H_{13}N_5O_4$	267.10	Adenosine	268.1051;136.0618;119.0345	All	58-61-7	997348	0.98%	√	
5	13.50	$[M+H]^+$	250.1437	250.1438	-0.3	$C_{14}H_{19}NO_3$	249.14	(2R,4R)-2-carboxy-1,1-dimethyl-4-(phenylmethoxy)-Pyrrolidinium	250.1432;84.0784;56.0466	/	1070772-59-6	2163810	2.13%		
6	13.81	$[M+H]^+$	250.1447	250.1438	3.7	$C_{14}H_{19}NO_3$	249.14	(2R,4R)-2-carboxy-1,1-dimethyl-4-(phenylmethoxy)-Pyrrolidinium isomer	250.1432;84.0784;56.0467	/	/	1499658	1.47%		
7	13.87	$[M-H]^-$	209.0446	209.0455	-4.5	$C_{10}H_{10}O_5$	210.05	(p-Hydroxybenzyl)malonic acid	165.0533;121.0629;93.0323;59.0113	Glycyrrhizae Radix et Rhizoma	90844-16-9	1615664	1.59%		1
8	17.98	$[M+H]^+$	565.1563	565.1552	2.0	$C_{26}H_{28}O_{14}$	564.15	Schaftoside	565.1589;547.1470;529.1365;427.1045;379.0840;325.0719	Glycyrrhizae Radix et Rhizoma	51938-32-0	2168819	2.13%	√	
9	19.26	$[M+H]^+$	579.1733	579.1708	4.3	$C_{27}H_{30}O_{14}$	578.16	Violanthin	579.1785;561.1672;543.1556;	Glycyrrhizae	40581-1	1975749	1.94%	√	

									5251420;457.1148;379.0803;325.0682	Radix et Rhizoma	7-7				
10	19.59	$[M-H]^-$	549.1649	549.1614	6.4	$C_{26}H_{30}O_{13}$	550.17	Liquiritin apioside	549.1618;255.0662;135.0091; 119.0502	Glycyrrhizae Radix et Rhizoma	74639-14-8	10971083	10.78%	√	
11	19.78	$[M-H]^-$	417.1196	417.1191	1.2	$C_{21}H_{22}O_9$	418.13	Liquiritin	255.0654;148.0193;135.0086; 119.0497;	Glycyrrhizae Radix et Rhizoma	551-15-5	11405831	11.21%	√	
12	21.97	$[M+H]^+$	679.5146	679.5117	4.3	$C_{36}H_{66}N_6O_6$	678.50	Cyclic hexaleucine	679.5183;661.5052;336.2317; 209.1657	All	83793-12-8	1667077	1.64%		
13	22.15	$[M-H]^-$	187.0974	187.0976	-1.0	$C_9H_{16}O_4$	188.10	Azelaic acid	187.0980;169.0867;125.0970; 97.0664	Zingiberis Rhlzoma	123-99-9	1163169	1.14%		2
14	23.01	$[M-H]^-$	549.1657	549.1614	7.9	$C_{26}H_{30}O_{13}$	550.17	Isoliquiritin apioside	549.1695;255.0679;135.0072; 119.0489	Glycyrrhizae Radix et Rhizoma	120926-46-7	1603404	1.58%	√	
15	23.53	$[M+H]^+$	431.1342	431.1337	1.3	$C_{22}H_{22}O_9$	430.13	Ononin	269.0806;254.0584;237.0545; 213.0871	Glycyrrhizae Radix et Rhizoma	486-62-4	3301338	3.24%	√	
16	23.65	$[M-H]^-$	417.1211	417.1191	4.8	$C_{21}H_{22}O_9$	418.13	Isoliquiritin	255.0670;148.0164;135.0071; 119.0487	Glycyrrhizae Radix et Rhizoma	5041-81-6	1748860	1.72%	√	
17	25.22	$[M-H]^-$	255.0670	255.0663	2.8	$C_{15}H_{12}O_4$	256.07	Liquiritigenin	255.0649;135.0069;119.0487; 91.0176	Glycyrrhizae Radix et Rhizoma	578-86-9	2691896	2.65%	√	
18	26.91	$[M-H]^-$	853.3985	853.4016	-3.6	$C_{42}H_{62}O_{18}$	854.39	22-Hydroxy-Licoricesaponin G2	853.3838;351.0562	Glycyrrhizae Radix et Rhizoma	1262326-48-6	623828	0.61%		3
19	27.21	$[M+H]^+$	985.4686	985.4639	4.8	$C_{48}H_{72}O_{21}$	984.46	Licoricesaponin A3	985.4847;809.4508;647.3904; 615.3991;453.3425	Glycyrrhizae Radix et Rhizoma	118325-22-7	2939612	2.89%		4
20	28.24	$[M+H]^+$	881.4210	881.4165	5.1	$C_{44}H_{64}O_{18}$	880.41	22β-Acetoxyl-glycyrrhizin	881.4352;705.3977;529.3568; 511.3481;451.3227	Glycyrrhizae Radix et Rhizoma	938042-17-2	1174216	1.15%		4
21	28.53	$[M+H]^+$	839.4083	839.4060	2.8	$C_{42}H_{62}O_{17}$	838.40	Macedonoside A	839.4206;663.3845;487.3462; 469.3363;451.3252	Glycyrrhizae Radix et Rhizoma	256441-31-3	1769702	1.74%		3

22	30.11	$[M+H]^+$	839.4134	839.4060	8.8	$C_{42}H_{62}O_{17}$	838.40	Licorice saponin G2	839.4220;663.3851;645.3725; 487.3455;469.3343;451.3233	Glycyrrhizae Radix et Rhizoma	118441-84-2	7669503	7.54%		4
23	30.75	$[M+H]^+$	839.4088	839.4060	3.4	$C_{42}H_{62}O_{17}$	838.40	Uralsaponin U	839.4216;663.3847;645.3731; 487.3464;469.3352;451.3247	Glycyrrhizae Radix et Rhizoma	1616062-86-2	2222775	2.18%		
24	31.14	$[M+H]^+$	839.4130	839.4060	8.4	$C_{42}H_{62}O_{17}$	838.40	Uralsaponin N	839.4190;645.3741;487.3488; 469.3368;451.3268	Glycyrrhizae Radix et Rhizoma	1616062-79-3	2901117	2.85%		4
25	31.26	$[M-H]^-$	821.4089	821.4118	-3.5	$C_{42}H_{62}O_{16}$	822.40	Glycyrrhizin	821.3986;351.0573;193.0362	Glycyrrhizae Radix et Rhizoma	1405-86-3	6638982	6.53%	√	
26	32.02	$[M-H]^-$	821.4071	821.4118	-5.7	$C_{42}H_{62}O_{16}$	822.40	Uralsaponin B	821.3990;351.0528;193.0319	Glycyrrhizae Radix et Rhizoma	105038-43-5	3194677	3.14%		5
27	32.26	$[M+H]^+$	823.4155	823.4111	5.4	$C_{42}H_{62}O_{16}$	822.40	Licoricesaponin H2	823.4234;647.3826;611.3652; 471.3510;453.3404;435.3289	Glycyrrhizae Radix et Rhizoma	118441-85-3	2040113	2.01%		
28	32.48	$[M+H]^+$	825.4300	825.4267	4.0	$C_{42}H_{64}O_{16}$	824.42	Licoricesaponin J2	825.4444;649.4079;613.3861; 455.3592;437.3481	Glycyrrhizae Radix et Rhizoma	938042-18-3	3076784	3.02%		
29	32.80	$[M-H_2O+H]^+$	277.1805	277.1803	0.6	$C_{17}H_{26}O_4$	294.18	6-Gingerol	177.0896;162.0685;145.0633; 137.0599;117.0689	Zingiberis Rhlzoma	23513-14-6	1352791	1.33%	√	
30	33.06	$[M-H]^-$	807.4237	807.4172	8.0	$C_{42}H_{64}O_{15}$	808.42	Licoricesaponin B2	807.4200;351.0538;193.0328	Glycyrrhizae Radix et Rhizoma	118536-86-0	786084	0.77%		
31	33.73	$[M+H]^+$	249.1491	249.1485	2.3	$C_{15}H_{20}O_3$	248.14	Atractylenolide III	231.1402;213.1285;189.0910; 163.0749;105.0683;91.0521	Macrocephalae Rhizoma	73030-71-4	101440	0.10%	√	
32	34.13	$[M+H]^+$	647.3835	647.3790	7.0	$C_{36}H_{54}O_{10}$	646.37	Glycyrrhetic acid 3-O-glucuronide	647.3877;471.3535;453.3415; 435.3311	Glycyrrhizae Radix et Rhizoma	34096-83-8	3438001	3.38%		
33	35.93	$[M+H]^+$	233.1541	233.1536	2.1	$C_{15}H_{20}O_2$	232.15	Atractylenolide Ⅱ	233.1551;215.1434;187.1479; 131.0853	Macrocephalae Rhizoma	73030-71-4	211276	0.21%	√	
34	36.37	$[M-H_2O+H]^+$	467.3168	467.3161	1.5	$C_{30}H_{44}O_5$	484.32	Poricoic acid B	467.3207;449.3101;325.2193; 307.2082;223.1480	Poria	137551-39-4	2969583	2.92%	√	

35	37.05	$[M-H]^-$	497.3312	497.3272	7.9	$C_{31}H_{46}O_5$	498.33	Poricoic acid A	497.3270;423.2910	Poria	137551-38-3	1126775	1.11%	√
36	37.23	$[M-H]^-$	485.3313	485.3272	8.3	$C_{30}H_{46}O_5$	486.33	Poricoic acid G	485.3302;441.3380;423.3327;397.2722	Poria	415724-84-4	794109	0.78%	
37	38.25	$[M+H]^+$	471.3491	471.3469	4.7	$C_{30}H_{46}O_4$	470.34	Glycyrrhetinic acid	471.3488;317.2089;235.1668;189.1619	Glycyrrhizae Radix et Rhizoma	526-95-4	1715150	1.69%	√

The source of the components was determined by the Natural Products HR-MS/MS Spectral Library 1.0 database and the literature.

GJLZ: Ganjianglingzhu.

Table S4. List of the identified metabolites of the intersection statistics between MCS vs. MCD and MCD vs. GL in positive and negative ion mode based on UHPLC-Q-TOF/MS

m/z	Retention time (min)	Ion mode	Formula	Metabolites	VIP	P-value	FC	
							MCS vs. MCD	MCD vs. GL
453.2860415	7.848233333	neg	C24H40O5	alpha-Muricholic acid	16.06769146	0.001366585	0.226549791	4.844474413
854.5920444	11.47483333	neg	C46H84NO8P	PE(22:4(7Z,10Z,13Z,16Z)/19:0)	11.85212432	0.000161857	1.861265702	0.653574112
373.2735342	8.718383333	pos	C24H36O3	3alpha-Hydroxy-5beta-chola-7,9(11)-dien-24-oic Acid	10.53291874	0.001324031	0.254796555	4.932638804
469.2808627	6.996783333	neg	C24H40O6	3alpha,6alpha,7beta,12alpha-Tetrahydroxy-5beta-cholan-24-oic Acid	9.722835203	0.00032875	0.108749729	5.89703359
786.6001094	12.6016	pos	C44H86NO9P	PS(O-20:0/18:1(9Z))	9.283616715	0.016021262	1.313988022	0.796299615
526.2928203	10.2723	pos	C27H44NO7P	LysoPE(0:0/22:6(4Z,7Z,10Z,13Z,16Z,19Z))	9.248142084	0.002656101	0.589762419	1.94542687
475.2678339	7.848233333	neg	C26H38O5	15-keto Latanoprost	7.181839741	0.001131022	0.312034456	3.641811108
837.5497715	7.848233333	neg	C43H83O13P	PI(18:0/16:0)	5.834670211	0.002426321	0.074756485	17.15141474
113.0713445	14.8042	pos	C5H5NO	2-Hydroxypyridine	5.509739735	7.52517E-06	1.075065689	0.845072861
165.113551	14.23996667	pos	C8H14N4O	Carcinine	5.38750534	0.000454935	1.071885203	0.879479787
778.5606782	11.15276667	neg	C40H80NO8P	PE(17:0/18:0)	5.172443046	2.76339E-05	1.490406477	0.463082293
817.5815506	7.860566667	pos	C47H83N2O8P	SM(d20:1/22:6(4Z,7Z,11E,13Z,15E,19Z)-2OH(10S,17))	4.934201668	0.004592119	0.063764653	20.246504
140.0818873	14.62146667	pos	C6H9N3O	4-Amino-5-hydroxymethyl-2-methylpyrimidine	4.893726991	6.10507E-05	1.085666755	0.865260905
528.3084425	10.55285	pos	C27H46NO7P	LysoPE(0:0/22:5(7Z,10Z,13Z,16Z,19Z))	4.815800027	0.001703836	0.636027276	2.437083044
136.0870209	14.23996667	pos	C7H11N3O	N-Acetylhistamine	4.534554115	0.0001237	1.078505749	0.873425882
385.2921892	11.77858333	pos	C20H42O5	Tetraethylene glycol monododecyl ether	4.336763663	5.92961E-06	1.14370385	0.789299844
167.1291583	13.75586667	pos	C8H11N3	N4-Cyclopropylpyridine-3,4-diamine	4.255261238	0.000544813	1.092627165	0.890206265
391.2838236	8.718383333	pos	C24H40O5	Cholic acid	4.141842644	0.001435452	0.346500761	2.984338756
140.1182257	13.57166667	pos	C7H10N2	2-Ethyl-3-methylpyrazine, 9CI	3.673824424	0.000215157	1.089113659	0.876178846
583.3128792	6.284083333	neg	C30H48O11	Cholic acid glucuronide	3.44490509	0.001491345	0.010451425	8.592156431

384.1151189	2.858283333	pos	C14H17N5O8	N6-Succinyl Adenosine	3.058379975	0.000341774	0.407844814	1.440757454
322.1068463	1.148183333	pos	C11H19N3O6S	Glycine, L-g-glutamyl-L-cysteinyl-, 3-methyl ester	3.014644757	2.8149E-05	2.721605591	0.549565878
531.2730091	13.22983333	neg	C25H43O7P	LysoPA(22:4(7Z,10Z,13Z,16Z)/0:0)	3.010902638	1.91644E-05	0.237951743	1.660520967
206.1401319	14.23996667	pos	C10H15N5	trapidil	2.926539149	0.000905415	1.114911152	0.91130873
219.1353051	14.00665	pos	C12H20O2	5-Dodecen-11-olide	2.855754692	5.05922E-05	1.063908597	0.852442627
627.2379316	6.996783333	neg	C40H38O8	Kuwanon V	2.739527784	0.000187906	0.113980576	5.895979673
250.0934556	2.0601	pos	C10H13N5O4	3'-Azido-3'-deoxythymidine, 98%	2.556042044	0.01805219	1.996704237	0.60146999
438.3790106	14.88846667	pos	C26H49D3O2	Cerotic acid(d3)	2.317113149	0.000779744	1.130871615	0.884973633
355.262961	6.936316667	pos	C24H36O3	(22E)-3beta-Hydroxy-5alpha-chola-7,22-dien-24-oic Acid	2.316396969	0.004799261	0.353599115	7.972818097
152.0819165	15.45048333	pos	C7H11N3O2	4-[(2R)-2-Aminopropyl]imidazole-1-carboxylic acid	2.315236581	3.19438E-05	1.071715431	0.857700825
469.3136183	12.68865	pos	C24H46O7	bhas#32	2.15548826	0.00030328	1.099248695	0.878286546
679.2300008	7.848233333	neg	C25H44O21	6''-O-alpha-D-Galactopyranosylciceritol	2.105023846	0.003114694	0.438025094	2.567048178
510.2532577	5.433816667	neg	C26H43NO8S	Glycochenodeoxycholate-3-sulfate	2.083568622	0.028446879	3.425319018	0.076758719
640.29266	11.58128333	neg	C30H47N3O10S	Lipoxin C4	2.051988399	0.012013848	0.627328289	1.369590968
1041.512013	7.848233333	neg	C52H86O18P2	PIP(22:3(10Z,13Z,16Z)/PGJ2)	2.017022227	0.00055419	0.124993695	11.39962287
303.2316966	11.92163333	pos	C20H30O2	8,13-Abietadien-18-oic acid	1.972066461	0.000450838	0.21527453	1.949930443
492.2580989	7.848233333	neg	C25H34F3N5O2	4-[[2-Methyl-2-[4-[5-(trifluoromethyl)pyridin-2-yl]piperazin-1-yl]propanoyl]amino]adamantane-1-carboxamide	1.875310612	0.000990815	0.22547304	4.310799004
469.2808552	6.097783333	neg	C24H40O6	1,3,7,12-Tetrahydroxycholan-24-oic acid	1.868118712	0.002301881	0.147336927	3.243054045
869.5404484	6.996783333	neg	C45H79NO10P+	PC(DiMe(9,3)/MonoMe(9,5))	1.826523141	0.002387582	0.03411912	41.30518855
205.1197046	14.23996667	pos	C9H12N6	Triethylenemelamine	1.775420506	2.27093E-05	1.062822884	0.843406864
443.2570897	8.724516667	neg	C23H41O6P	sn-3-O-(geranylgeranyl)glycerol 1-phosphate	1.734620937	0.000615875	0.169564818	6.888963795
763.213171	6.996783333	neg	C19H18N4O3S	Ilaprazole sulfone	1.682270911	9.99513E-05	0.107766847	6.579491374
1045.537204	8.724516667	neg	C34H37NO4	Deoxynojirimycin Tetrabenzyl Ether	1.661775502	0.000587262	0.245658693	7.25788903
450.0882438	2.856116667	neg	C17H25NO9S2	4-Phenylbutyl glucosinolate	1.628887677	0.000676201	0.421192865	1.607825373

136.0619124	0.729716667	pos	C5H7N5O	FAPy-adenine	1.588370802	0.003613695	2.044405623	0.675213054
837.5503184	7.605466667	neg	C46H81NO10P+	PC(MonoMe(9,5)/MonoMe(9,5))	1.584641504	0.008909771	0.044907589	6.119643283
552.3673712	11.15276667	neg	C26H54NO6P	LysoPC(P-18:0/0:0)	1.543319381	0.000198229	0.33215501	1.575619141
928.5596231	7.866566667	neg	C47H82NO12P	PE(DiMe(11,5)/PGE1)	1.524933185	0.006515024	0.296557416	3.248489144
407.280584	7.113833333	neg	C24H40O5	Ursocholic acid	1.51902355	0.012736016	0.425447154	2.538338262
590.1259756	1.042583333	neg	C27H29O16-	kaempferol 3-O-beta-D-glucosyl-(1->2)-glucoside	1.506139118	5.2285E-06	0.213989725	1.571514411
431.2770534	7.841416667	pos	C24H40O5	3a,7b,12b-Trihydroxy-5b-cholanoic acid	1.486833468	0.00297024	0.181847837	6.591262344
249.1820697	13.75586667	pos	C12H17N5	N-Cyano-N'-(1,1-dimethylpropyl)-N''-(3-pyridinyl)guanidine	1.473713124	0.000707456	1.082650965	0.88653613
499.2640293	8.734933333	pos	C28H44O3S	(6R)-vitamin D2 6,19-sulfur dioxide adduct	1.461883188	0.001245877	0.290850607	4.658965578
1109.498821	7.848233333	neg	C50H80O24	12-Ketoporrigenin 3-[4'-(2''-glucosyl-3''-xylosyl)-glucosyl)-galactoside]	1.454907288	0.000315361	0.135750355	10.5948682
163.1158283	0.665216667	pos	C8H18OS	(S1)-Methoxy-3-heptanethiol	1.447537942	0.020152273	1.781726721	0.735144
831.2007612	6.996783333	neg	C37H38O19	Isoorientin 4'-O-glucoside 2''-O-(E)-ferulate	1.446845535	8.74017E-05	0.11331658	6.91509619
193.1587503	8.007083333	pos	C13H20O	(E)-5,8-Megastigmadien-4-one	1.430111065	1.58911E-06	1.264781237	0.67808904
528.3097809	10.75313333	neg	C27H48NO7P	LysoPE(22:4(7Z,10Z,13Z,16Z)/0:0)	1.418176566	0.012753014	0.584093348	2.126418034
124.051392	14.38935	pos	C5H5N3O	Pyrazinamide	1.407028631	5.73176E-05	1.094912955	0.856642992
306.1553507	0.7808	pos	C12H21N5O3	Histidinyl-Lysine	1.356728026	0.034405093	2.693734274	0.653849062
136.0619968	1.111166667	pos	C5H5N5	1H-Pyrazolo[3,4-d]pyrimidin-4-amine	1.347558849	0.028379399	1.432794521	0.792946992
747.2181513	8.743216667	neg	C18H18N2O7	Harmol glucuronide	1.332297402	0.00105241	0.253479508	5.661285363
719.2879001	6.284083333	neg	C19H21FN2O4	Nadifloxacin	1.291575671	0.002116306	0.004885268	7.906607627
211.1692916	8.0247	pos	C13H24O3	11-Hydroxy-9-tridecenoic acid	1.279796576	3.14491E-06	1.331104455	0.683194107
268.1880893	8.007083333	pos	C15H25NO3	Metoprolol	1.255003724	8.86353E-06	1.486426442	0.640754463
937.5277516	6.996783333	neg	C45H81O15P	PI(20:3(8Z,11Z,14Z)-2OH(5,6)/16:0)	1.251376505	0.00181594	0.033071913	64.86121275
1117.548992	8.707483333	neg	C31H37N5O5	Benzamide, N-[2-[4-(cyclopropylcarbonyl)-3-methyl-1-piperazinyl]-1-[[7-(1,1-dimethylethyl)-1H-indol-3-yl]methyl]-2-oxoethyl]-4-nitro-	1.240800633	0.003363721	1.68622979	0.525468413

236.1619925	7.088483333	pos	C12H23NO2	N-(9-Oxodecyl)acetamide	1.182644386	0.000212536	1.409477864	0.739531969
922.5276174	7.848233333	neg	C44H80NO14P	PS(18:0/TXB2)	1.129564766	0.000799651	0.066189535	17.64649025
447.2716214	6.991983333	pos	C22H31N5O4	Melagatran	1.118092303	0.000468984	0.128608479	5.856580276
928.565239	8.718383333	pos	C45H84O14P2	PGP(18:3(9,11,15)-OH(13)/i-21:0)	1.108556196	0.00109309	0.277291349	5.805766627
483.2962839	9.099666667	neg	C25H42O6	3beta-(3-methyl-butanoyloxy)-villanovane-13alpha,17-diol	1.106592904	0.000429405	0.120032802	12.77642541
195.1605142	13.47035	pos	C10H15N3	Bethanidine	1.105820183	0.001409466	1.107611457	0.90868718
570.2565103	10.2723	pos	C27H36O12	Prupaside	1.067156736	0.001465246	0.569496573	1.535973568
815.2052669	8.743216667	neg	C19H20O10	Khellol glucoside	1.057623249	0.000232045	0.192279012	7.416713369
576.2409991	6.996783333	neg	C26H36F3NO7	Fluprostenol serinol amide	1.046094961	0.000213377	0.102580001	6.018989931
905.537942	7.605466667	neg	C52H76O10	(9Z)-Zeaxanthin dirhamnoside	1.042103184	0.007786404	0.04170917	6.402904743
263.1614072	13.75586667	pos	C12H15N5O	[(1R,5R)-5-(6-Aminopurin-9-yl)cyclohex-3-en-1-yl]methanol	1.029866284	0.000111848	1.119024884	0.89300329

FC: fold change; GJLZ: Ganjianglingzhu; GL: low dose of GJLZ; MCD: methionine-choline-deficient; MCS: methionine-choline-sufficient; VIP: variable importance of projection.

Table S5. The correlation between biochemical indicators and the metabolites

	TC	TG	HDL-C	ALT	AST	FBG	TBIL	SOD	T-AOC	MDA
alpha-Muricholic acid	-0.164	.558*	-0.386	0.362	.600**	0.015	0.19	-.625**	-0.34	.522*
PE(22:4(7Z,10Z,13Z,16Z)/19:0)	0.489	-.562*	.750**	-.585*	-.616**	0.377	-0.479	.520*	.575**	-.561*
3alpha-Hydroxy-5beta-chola-7,9(11)-dien-24-oic Acid	-0.089	.600*	-0.421	0.476	.608**	-0.027	0.235	-.583*	-0.361	.581*
3alpha,6alpha,7beta,12alpha-Tetrahydroxy-5beta-cholan-24-oic Acid	-0.421	.591*	-.582*	0.403	.628**	-0.222	0.361	-.694**	-.493*	.681**
PS(O-20:0/18:1(9Z))	0.446	-0.345	.718**	-.738**	-.775**	0.243	0.036	0.451	.747**	-0.365
LysoPE(0:0/22:6(4Z,7Z,10Z,13Z,16Z,19Z))	-0.239	.543*	-0.429	.506*	.581*	-0.18	0.005	-.596*	-.695**	0.36
15-keto Latanoprost	-0.175	.530*	-0.386	0.341	.593**	0.02	0.174	-.615**	-0.325	.507*
PI(18:0/16:0)	-0.132	.580*	-0.357	0.35	.591**	0.02	0.18	-.615**	-0.354	.510*
2-Hydroxypyridine	0.05	-.508*	0.2	-0.25	-0.17	0.084	-0.372	0.321	0.344	-0.257
Carcinine	0.014	-.488*	0.161	-0.353	-0.183	0.124	-0.417	0.301	0.328	-0.252
PE(17:0/18:0)	0.171	-.585*	0.186	-0.229	-0.174	-0.004	-0.242	0.252	0.412	-0.164
SM(d20:1/22:6(4Z,7Z,11E,13Z,15E,19Z)-2OH(10S,17))	-0.161	.603*	-0.382	0.418	.633**	0.059	0.131	-.627**	-0.404	0.478
4-Amino-5-hydroxymethyl-2-methylpyrimidine	-0.082	-.602*	0.2	-0.235	-0.234	0.128	-0.445	0.333	0.335	-0.257
LysoPE(0:0/22:5(7Z,10Z,13Z,16Z,19Z))	-0.157	.645**	-0.321	.553*	0.451	0.011	0.321	-0.355	-.475*	0.279
N-Acetylhistamine	0.025	-.494*	0.189	-0.359	-0.203	0.136	-0.379	0.282	0.384	-0.216
Tetraethylene glycol monododecyl ether	-0.043	-.697**	0.171	-0.176	-0.189	0.206	-0.242	0.346	.533*	-0.221
N4-Cyclopropylpyridine-3,4-diamine	0.146	-.489*	0.311	-0.315	-0.247	0.323	-0.418	0.38	.484*	-0.377
Cholic acid	-0.161	.551*	-0.489	0.429	.589*	-0.124	0.332	-.630**	-0.382	.632**
2-Ethyl-3-methylpyrazine, 9CI	0.104	-0.47	0.311	-0.326	-0.238	0.234	-0.384	0.353	.470*	-0.304
Cholic acid glucuronide	-.660**	.503*	-.839**	.726**	.768**	-0.375	0.463	-.769**	-.575*	.762**
N6-Succinyl Adenosine	-0.432	0.303	-.679**	0.265	0.41	-.609**	0.485	-.713**	-0.398	.716**
Glycine, L-g-glutamyl-L-cysteinyl-, 3-methyl ester	.782**	-0.408	.875**	-.559*	-.752**	0.408	-0.226	.672**	.595**	-.669**
LysoPA(22:4(7Z,10Z,13Z,16Z)/0:0)	-.550*	0.433	-.782**	.624**	.688**	-.711**	0.264	-.792**	-.758**	.841**

trapidil	0.321	-0.374	0.432	-0.365	-0.296	.522*	-.598*	.527*	.482*	-.556*
5-Dodecen-11-olide	0.021	-.516*	0.207	-0.312	-0.199	0.111	-0.479	0.341	0.349	-0.265
Kuwanon V	-0.482	.565*	-.611*	0.468	.639**	-0.253	0.361	-.711**	-.525*	.718**
3'-Azido-3'-deoxythymidine, 98%	0.3	-.690**	0.5	-.512*	-0.465	0.306	-.528*	0.343	.526*	-0.402
Cerotic acid(d3)	0.432	-0.465	.546*	-0.385	-0.35	.504*	-0.422	0.343	.509*	-.566*
(22E)-3beta-Hydroxy-5alpha-chola-7,22-dien-24-oic Acid	-0.129	.660**	-0.311	.538*	.484*	0.105	0.352	-0.426	-0.295	0.441
4-[(2R)-2-Aminopropyl]imidazole-1-carboxylic acid	0.061	-.484*	0.182	-0.3	-0.166	0.098	-0.427	0.326	0.351	-0.248
bhas#32	0.282	-0.389	0.382	-0.25	-0.269	0.391	-0.332	.534*	.565*	-0.463
6''-O-alpha-D-Galactopyranosylciceritol	-0.139	.503*	-0.386	0.274	.556*	0.053	0.131	-.569*	-0.291	0.453
Glycochenodeoxycholate-3-sulfate	0.279	-0.372	0.325	-0.253	-0.236	0.163	-0.33	0.463	.458*	-0.289
Lipoxin C4	-0.371	0.319	-.689**	0.447	.670**	-0.394	0.018	-.559*	-.684**	0.395
PIP(22:3(10Z,13Z,16Z)/PGJ2)	-0.115	.568*	-0.326	0.304	.544*	0.056	0.188	-.582*	-0.338	0.455
8,13-Abietadien-18-oic acid	-.543*	.521*	-.768**	.674**	.690**	-.567**	0.422	-.608**	-.761**	.762**
4-[[2-Methyl-2-[4-[5-(trifluoromethyl)pyridin-2-yl]piperazin-1-yl]propanoyl]amino]adamantane-1-carboxamide	-0.243	.560*	-0.432	0.374	.624**	0.014	0.189	-.618**	-0.361	.525*
1,3,7,12-Tetrahydroxycholan-24-oic acid	-0.332	0.46	-.518*	0.365	.552*	-0.167	0.309	-.551*	-0.398	.667**
PC(DiMe(9,3)/MonoMe(9,5))	-0.483	.548*	-.568*	0.446	.654**	-0.159	0.271	-.703**	-.487*	.663**
Triethylenemelamine	0.061	-.494*	0.211	-0.371	-0.236	0.063	-0.413	0.324	0.372	-0.277
sn-3-O-(geranylgeranyl)glycerol 1-phosphate	-0.189	.560*	-0.443	0.475	.569*	-0.053	0.29	-.624**	-0.354	.649**
Ilaprazole sulfone	-0.47	.574*	-.616*	0.472	.650**	-0.248	0.386	-.715**	-.520*	.710**
Deoxynojirimycin Tetrabenzyl Ether	-0.041	.576*	-0.288	0.427	.491*	0.067	0.274	-.584*	-0.255	.520*
4-Phenylbutyl glucosinolate	-0.339	0.327	-.579*	0.209	0.375	-.562**	.580*	-.696**	-0.384	.706**
FAPy-adenine	0.371	-.571*	.582*	-.506*	-.476*	.562**	-0.488	0.475	.616**	-.627**
PC(MonoMe(9,5)/MonoMe(9,5))	-0.204	0.398	-.516*	.564*	.706**	0.062	0.181	-.622**	-0.301	.504*
LysoPC(P-18:0/0:0)	-.668**	0.362	-.896**	.741**	.800**	-.609**	0.293	-.706**	-.737**	.738**
PE(DiMe(11,5)/PGE1)	-0.186	.645**	-0.332	0.465	.637**	0.06	-0.065	-.608**	-0.426	0.424

Ursocholic acid	-0.182	.575*	-0.421	.506*	.620**	0.167	0.095	-0.373	-0.295	0.453
kaempferol 3-O-beta-D-glucosyl-(1->2)-glucoside	-.611*	0.44	-.843**	.768**	.750**	-.618**	.517*	-.610**	-.704**	.755**
3a,7b,12b-Trihydroxy-5b-cholanoic acid	-0.1	.549*	-0.311	0.326	.581*	0.072	0.07	-.647**	-0.332	.498*
N-Cyano-N'-(1,1-dimethylpropyl)-N''-(3-pyridinyl)guanidine	0.064	-0.456	0.25	-0.285	-0.179	0.246	-0.433	0.387	0.416	-0.368
(6R)-vitamin D2 6,19-sulfur dioxide adduct	-0.071	.625**	-0.411	0.479	.577*	-0.043	0.352	-.583*	-0.354	.574*
12-Ketoporrigenin 3-[4'-(2''-glucosyl-3''-xylosyl)-glucosyl)-galactoside]	-0.172	.539*	-0.369	0.298	.560*	0.016	0.203	-.617**	-0.367	.526*
(S1)-Methoxy-3-heptanethiol	.557*	-0.161	.629*	-0.474	-.600**	0.251	0.147	.642**	.630**	-.488*
Isoorientin 4'-O-glucoside 2''-O-(E)-ferulate	-0.455	.565*	-.595*	0.416	.628**	-0.226	0.359	-.698**	-.501*	.695**
(E)-5,8-Megastigmadien-4-one	0.218	-.660**	0.411	-.529*	-.494*	0.056	-0.232	0.336	.458*	-0.252
LysoPE(22:4(7Z,10Z,13Z,16Z)/0:0)	-0.121	.495*	-0.343	.576*	0.414	-0.183	0.206	-0.137	-.554*	0.093
Pyrazinamide	0.068	-.558*	0.221	-0.362	-0.226	0.243	-0.408	0.387	.456*	-0.287
Histidinyl-Lysine	.525*	0.011	.579*	-0.321	-0.276	0.358	-0.174	0.471	0.426	-0.277
1H-Pyrazolo[3,4-d]pyrimidin-4-amine	0.311	-.488*	0.454	-.535*	-0.424	.477*	-0.474	0.338	.458*	-.532*
Harmol glucuronide	-0.09	.627**	-0.401	.513*	.562*	-0.001	0.374	-.560*	-0.315	.510*
Nadifloxacin	-.693**	0.363	-.835**	.678**	.758**	-0.398	0.373	-.815**	-.548*	.760**
11-Hydroxy-9-tridecenoic acid	0.289	-.704**	.536*	-.659**	-.589*	0.167	-0.269	0.365	.525*	-0.365
Metoprolol	0.364	-.625**	.629*	-.724**	-.645**	0.21	-0.303	0.419	.532*	-0.417
PI(20:3(8Z,11Z,14Z)-2OH(5,6)/16:0)	-0.483	.548*	-.568*	0.446	.654**	-0.161	0.271	-.703**	-.488*	.663**
Benzamide, N-[2-[4-(cyclopropylcarbonyl)-3-methyl-1-piperazinyl]-1-[[7-(1,1-dimethylethyl)-1H-indol-3-yl]methyl]-2-oxoethyl]-4-nitro-	-0.104	-.652**	0.239	-0.265	-0.414	0.014	-0.338	0.439	0.251	-0.458
N-(9-Oxodecyl)acetamide	0.343	-.537*	.643**	-.718**	-.602**	0.253	-0.427	.498*	0.446	-.517*
PS(18:0/TXB2)	-0.168	.508*	-0.385	0.307	.550*	-0.1	0.246	-.733**	-0.425	.581*
Melagatran	-0.419	.570*	-.566*	0.428	.607**	-0.187	0.405	-.676**	-.467*	.666**
PGP(18:3(9,11,15)-OH(13)/i-21:0)	-0.139	.579*	-0.421	.530*	.597**	-0.009	0.379	-.611**	-0.351	.560*
3beta-(3-methyl-butanoyloxy)-villanovane-13alpha,17-diol	-0.389	.628**	-.573*	.513*	.677**	-0.102	0.269	-.655**	-.506*	.670**

Bethanidine	0.375	-0.408	0.496	-0.403	-0.366	0.441	-0.397	0.453	.553*	-.537*
Prupaside	-0.468	0.456	-.689**	.653**	.783**	-0.355	-0.002	-.738**	-.739**	.574*
Khellol glucoside	-0.12	.541*	-0.393	0.474	.558*	-0.046	0.318	-.618**	-0.352	.548*
Fluprostenol serinol amide	-0.436	.584*	-.579*	0.4	.618**	-0.228	0.338	-.691**	-.498*	.691**
(9Z)-Zeaxanthin dirhamnoside	-0.189	0.363	-.530*	.573*	.704**	0.038	0.179	-.608**	-0.276	.512*
[(1R,5R)-5-(6-Aminopurin-9-yl)cyclohex-3-en-1-yl]methanol	0.321	-0.276	.525*	-0.332	-0.337	.519*	-0.424	.630**	.554*	-.547*

ALT: alanine transaminase; AST: aspartate transaminase; FBG: fasting blood glucose; HDL-C: high-density lipoprotein cholesterol; MDA: malondialdehyde; SOD: superoxide dismutase; TBIL: total bilirubin; TC: total cholesterol; TG: triglyceride; T-AOC: total antioxidant capacity. * Correlation is significant at the 0.05 level (2-tailed); ** correlation is significant at the 0.01 level (2-tailed).

References

1 Li, G., Nikolic, D. & van Breemen, R. B. Identification and Chemical Standardization of Licorice Raw Materials and Dietary Supplements Using UHPLC-MS/MS. *J Agric Food Chem* **64**, 8062-8070, doi:10.1021/acs.jafc.6b02954 (2016).

2 Xue, G. *et al.* Integrative analyses of widely targeted metabolomic profiling and derivatization-based LC-MS/MS reveals metabolic changes of Zingiberis Rhizoma and its processed products. *Food Chem* **389**, 133068, doi:10.1016/j.foodchem.2022.133068 (2022).

3 Song, L. *et al.* Structural identification and comprehensive comparison of saponin-related impurities present in the three different compound glycyrrhizin tablets. *J Pharm Biomed Anal* **229**, 115287, doi:10.1016/j.jpba.2023.115287 (2023).

4 Sun, L. *et al.* Rapid characterization of chemical constituents of Shaoyao Gancao decoction using UHPLC coupled with Fourier transform ion cyclotron resonance mass spectrometry. *RSC Adv* **10**, 29528-29535, doi:10.1039/d0ra04701e (2020).

5 Wong, Y. F. *et al.* Untargeted profiling of Glycyrrhiza glabra extract with comprehensive two-dimensional liquid chromatography-mass spectrometry using multi-segmented shift gradients in the second dimension: Expanding the metabolic coverage. *Electrophoresis*, doi:10.1002/elps.201700469 (2018).
